# Formation and stabilization of the telomeric antiparallel G-quadruplex and inhibition of telomerase by novel benzothioxanthene derivatives with anti-tumor activity

**DOI:** 10.1038/srep13693

**Published:** 2015-09-02

**Authors:** Wen Zhang, Min Chen, Yan Ling Wu, Yoshimasa Tanaka, Yan Juan Ji, Su Lin Zhang, Chuan He Wei, Yan Xu

**Affiliations:** 1 Lab of Chemical Biology and Molecular Drug Design, College of Pharmaceutical Science, Zhejiang University of Technology, 18 Chaowang Road, Hangzhou, 310014, China; 2 Lab of Molecular Immunology, Zhejiang Provincial Center for Disease Control and Prevention, 3399 Binsheng Road, Hangzhou, 310051, China; 3 Center for Innovation in Immunoregulative Technology and Therapeutics, Graduate School of Medicine, Kyoto University, Kyoto, 606-8501, Japan; 4 Division of Chemistry, Department of Medical Sciences, Faculty of Medicine, University of Miyazaki, 5200 Kihara, Kiyotake, Miyazaki, 889-1692, Japan

## Abstract

G-quadruplexes formed in telomeric DNA sequences at human chromosome ends can be a novel target for the development of therapeutics for the treatment of cancer patients. Herein, we examined the ability of six novel benzothioxanthene derivatives **S1**–**S6** to induce the formation of and stabilize an antiparallel G-quadruplex by EMSA, UV-melting and CD techniques and the influence of **S1**–**S6** on A549 and SGC7901 cells through real-time cell analysis, wound healing, trap assay methods. Results show that six compounds could differentially induce 26 nt G-rich oligonucleotides to form the G-quadruplex with high selectivity *vs* C-rich DNA, mutated DNA and double-stranded DNA, stabilize it with high affinity, promote apoptosis and inhibit mobility and telomerase activity of A549 cells and SGC7901 cells. Especially, **S1**, **S3**, **S4** displayed stronger abilities, of which **S3** was the most optimal with the maximum ΔT_m_ value being up to 29.8 °C for G-quadruplex, the minimum IC_50_ value being 0.53 μM and the maximum cell inhibitory rate being up to 97.2%. This study suggests that this type of compounds that induce the formation of and stabilize the telomeric antiparallel G-quadruplex, and consequently inhibit telomerase activity, leading to cell apoptosis, can be screened for the discovery of novel antitumor therapeutics.

Human telomeres are composed of tandem repeats of the short DNA motif, TTAGGG, and an array of telomeric proteins, and are involved in the protection of chromosome from deterioration and end-to-end fusion^1^,^2^. Telomeres can be divided into two functional regions, the double-stranded telomeric DNA and the single-stranded telomeric 3′-overhang^3^,^4^. One of the telomeric proteins termed protection of telomeres 1 (POT1) interacts with the telomeric 3′-overhang and modulates the activity of telomerase, the enzyme responsible for the extension of telomeres and the infinite proliferative capacity of tumor cells^5^,^6^,^7^,^8^. When the telomeric 3′-overhang is folded into G-quadruplexes, which can be influenced by cations, DNA concentration, pH, temperature, and ligands, POT1 can no longer interact with the telomeres, limiting the activity of telomerase. The formation and stabilization of G-quadruplexes are thus inextricably linked with the development of anticancer therapeutics. In addition to telomeric G-rich tracts, DNA and RNA sequences, such as proto-oncogenes, promoter regions, and immunoglobulin switch regions, can form intramolecular and intermolecular G-quadruplex structures, further justifying the strategy for developing anticancer therapeutics by screening inducers and/or stabilizers of G-quadruplexes^9^,^10^,^11^,^12^. It was reported that formation and stabilization of G-quadruplex structures by binding molecules can indirectly inhibit telomerase activity *in vitro*^13^,^14^. For example, telomestatin, a natural product that potently interacts with and stabilizes G-quadruplexes, induced apoptosis in cancer cells by uncapping POT1 from telomeres^15^,^16^. Accordingly, interaction of small molecules with G-quadruplex structures has been considered as an approach to design and develop new anticancer drugs^14^,^17^,^18^.

Our previous studies showed that benzoxanthene derivatives containing 1,8-naphthalimide ring can interact with G-rich telomere sequences and inhibit human telomerase activity (unpublished data), and also other groups have demonstrated that the 1,8-naphthalimides, as precursors of benzothioxanthenes, and their derivatives and analogues can interact with double-stranded DNA^19^,^20^ and G-quadrplexes^21^,^22^ with high affinity through π-π stacking mode, showing strong anticancer activities. However, benzoxanthenes with naphthalimido aromatic nucleus alone and 1,8-naphthalimides have poor water solubility and selectivity between G-quadruplex and double-strands DNA. In order to address these issues, we further optimized and modified the structure of this kind of compounds, and synthesized six novel benzo[k,l]thioxanthene-3,4-dicarboximides (benzothioxanthene derivatives) **S1**–**S6** (Fig. 1) with six kinds of protonated side chains which are optimum three carbon atoms-containing lengths sufficient to bind to G-quadruplex grooves^22^ as G-quadruplex targeted ligands. Introduction of these side chains to the benzothioxanthene mother structure is expected not only to increase hydropholicity, but also to effectively stabilize the G-quadruplex structure through the cationic functional groups directing toward the G-quadruplex grooves and then interacting with negatively charged phosphate groups^23^. Consequently these benzothioxanthene derivatives should have huge potential to stabilize G-quadruplex with enhanced specificity and anti-cancer activity by indirect inhibition of telomerase activity. Herein, we studied the interaction of **S1**–**S6** with 26 nt telomeric DNAs (Table 1), including guanine-rich DNA (G-rich DNA), cytosine-rich DNA (C-rich DNA), double-stranded DNA (ds-DNA) and Mutated DNA (Mut-DNA), by electrophoretic mobility shift assay (EMSA), UV thermal melting and circular dichroism (CD) techniques to monitor the ability of the six compounds to induce the formation of and to stabilize G-quadruplexes formed in the examined DNAs and assessed their biological activities through real-time cell analysis (RTCA), trap assay and wound healing to two kinds of cancer cells, A549 and SGC7901 which both express telomerase.

## Results

### Promotion of G-quadruplex formation by benzothioxanthene derivatives

**Figure 1:**
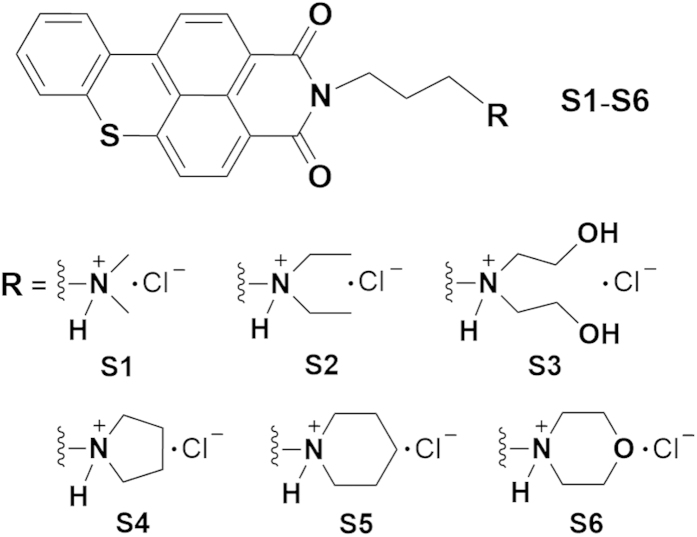
Structure of six novel benzo[k,l]thioxanthene-3,4-dicarboximides (S1–S6).

Structures of newly synthesized benzothioxanthene derivatives, **S1–S6**, and their synthesis were shown in Fig. 1 and Fig. S1, respectively, and the sequences of newly designed 26 nt telomeric DNAs for G-quadruplex formation assay were listed in Table 1. To measure the ability of **S1–S6** to induce the formation of and stabilize G-quadruplexes from the 26 nt telomeric DNA sequences, including the G-rich DNA containing four three-guanine repeats which has the potential to form G-quadruplexes, ds-DNA, C-rich DNA, and Mut-DNA completely impossible to form G-quadruplex structures in Tris buffer (10 mM Tris–HCl, 10 mM KCl, 0.1 mM EDTA, pH = 7.4), we examined the oligonucleotides treated with a serial dilution of compounds **S1**–**S6** for the formation of G-quadruplexes by means of EMSA using a Bio-Rad imaging detector.

**Figure 2:**
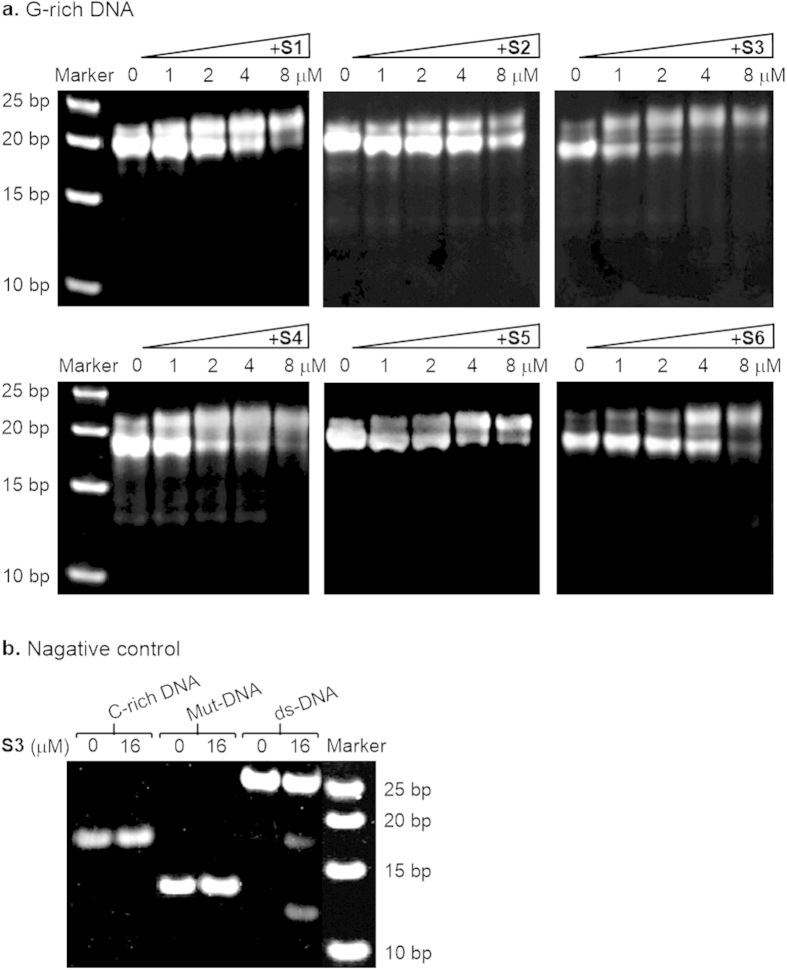
The ability of six benzothioxanthene derivatives to promote the formation of G-quadruplex structure in 26 nt telomeric DNA sequences. (**a**) EMSA images of Tel26nt-G-rich DNA in the presence and absence of S1–S6 at the concentration of 0, 2, 4 and 8 μM, increasing from left to right. (**b**) Negative control. Tel26nt-C-rich DNA, Tel26nt-double-stranded DNA and Tel26nt-mutated-strand DNA were used as a negative control and the S3 concentration was 0 and 16 μM. All experiments were performed in a buffer (pH 7.4) containing 10 mM Tris–HCl, 10 mM KCl, 0.1 mM EDTA, and samples of 2 μM DNA dissolved in the buffer in the presence and absence of compounds were incubated for 24 h after annealing at 95 °C. The reaction mixtures were resolved by electrophoresis (20% native polyacrylamide gel, 150 V, 4 h) and visualized by means of SYBR GOLD staining. Base pairs for used Marker are marked on the left side (**a**) and on the right side (**b**). Pre-preparation for all samples were shown in Methods section.

As shown in Fig. 2a, there exist at least two species of G-quadruplex conformations formed in G-rich DNA alone in gels, with one bright band migrating faster and the other very faint band migrating slower, which both may be ascribed to intermolecular G-quadruplexes relative to a double-stranded Marker on the left side in each gel^24^,^25^, but further determination of precise molecular structures are required by high-resolution techniques such as nuclear magnetic resonance (NMR) and x-ray crystallography. With increasing concentration of compounds, the slower-moving species distinctly gradually increased in a compound concentration-dependent manner and its increased amount and rate displayed different with addition of different compounds, suggesting that these compounds could differentially promote the conversion of the faster-migrating structure to the slower-migrating one and the formation of a novel compound-G-quadruplex complex, in particular, so very quickly and notably did **S1**, **S3**, and **S4**, of which **S3** was the strongest inducer and stabilizer to the slower-migrating G-quadruplex, even at 4 μM the faster-migrating structure was almost completely converted to the slower-migrating one. In addition, the band mobility for control oligonucleotides, including C-rich DNA, Mut-DNA, and ds-DNA was also observed in the presence and absence of **S3** in Fig. 2b, results showing that **S3** had no effect on the first two kinds of DNAs, even at a higher concentration of 16 μM, while a little on ds-DNA. Of those the 26 nt C-rich DNA as a complementary strand of G-rich DNA migrated a little slowly relative to a double-stranded DNA Marker on the right side in the gel shown in Fig. 2b, suggesting that it possibly adopted a freedom coil form, not a completely linear form; whereas Mut-DNA with a single G-to-C mutation introduced in the middle of runs of three guanines, impossible to form any G-quadruplex, and ds-DNA displayed normal mobility behaviors relative to the Marker. Other compounds presented similar results having no effects on three control oligonucleotides (data not shown).

Taken together, these data demonstrate that benzothioxanthene derivatives, especially **S1**, **S3, S4**, could specifically recognize the telomeric G-rich DNA sequence and promote the formation of and stabilize a species of G-quadruplex structure.

### Specific binding of benzothioxanthene derivatives to G-quadruplex structures

Melting curves are widely used to determine thermodynamic properties of folded nucleic acid structures including their stability and interaction with ligands^26^,^27^. The melting curve of duplex nucleic acids is generally closely related to a hyperchromic shift at 260 nm^28^, while the melting of G-quadruplex is associated with a hypochromic shift at 295 nm^29^. Herein, in order to assess binding affinity of benzothioxanthene derivatives with 26 nt telomeric DNAs, we monitored the UV absorbance of the nucleic acids as a function of temperature and then obtained the melting temperature (*Tm*, °C) that is the mid-point of a melting curve at which the complex is 50% dissociated for quadruplex-forming oligonucleotides in the presence and absence of compounds **S1–S6**. As shown in Figs S2A−F and Table 2, the absorbance of the G-rich DNA decreased with increasing the temperature, with a standard inverted “S” curve presented, characteristic of a G-quadruplex structure, and the ΔT_m_ values, relative to the *Tm* (38.5 °C) of the G-quadruplex alone, increased with increasing the concentration of **S1**–**S6**, demonstrating that **S1**–**S6** stabilized G-quadruplex structures. More it is worth noting that ΔT_m_s for all compounds a little increased at more than 2:1 rate of compound/DNA, but **S3** showing a little increase in ΔT_m_ at more than 4:1 rate, indicating that there exists 2:1 and 4:1 stoichiometry of **S1**-, **S2**-, **S4**-, **S5**-, **S6-**, and **S3**-DNA complexes, respectively; in addition, compounds **S1**, **S3**, **S4** produced more increases in ΔT_m_s of compound-G-quadruplex complexes, especially **S3** with maximum ΔT_m_ being up to 27 °C at the 4:1 rate, which supports the observations by EMSA. In contrast, essentially no effect of **S1**–**S6** was observed in the absorbance of control DNAs at the 8:1 ratio of compound/DNA as shown in Figs S3A−C, showing the specific binding of **S1**–**S6** to G-quadruplexes. To sum up, the EMSA and UV-melting studies both suggest that benzothioxanthene derivatives possess high selectivity and excellent inducing and stabilizing capability to the G-quadruplex structure rather than ds-DNA, and single-stranded DNAs that can not form G-quadruplexes.

### Benzothioxanthene derivatives promote the formation of antiparallel G-quadruplexes from the Tel26nt oligonucleotides

To in details study most properties of nucleic acids structures, apart from X-ray diffraction or NMR spectroscopy, circular dichroism (CD) spectroscopy is regard as the third regularly used method^30^,^31^,^32^. In particular, recently, CD spectroscopy, often as a pioneering approach, has extensively been applied in research on G-quadruplexes formed in DNA and RNA sequences, which can reveal the formation of G-quadruplex structures, the conditions stabilizing the structures, the transitions between various structural states, kinetics of their appearance, and features of ligand binding G-quadruplex^32^,^33^. In this study, we employed the CD technique to make attempt to further illuminate some interaction characteristics of benzothioxanthene derivatives with 26 nt telomeric DNAs (G-rich DNA, C-rich DNA and ds-DNA) and Mut-DNA (Table 1), including the G-quadruplex structures formed by 26 nt telomeric DNAs, their interaction modes, stoichiometry of compound-DNA complexes and so on^25^,^32^,^33^,^34^,^35^. Of six novel benzothioxanthene derivatives, **S1**, **S3** and **S4** producing stronger affinity to 26 nt telomeric G-rich DNA in EMSA and UV-melting experiments are representatives used in this study (Fig. 1).

**Figure 3:**
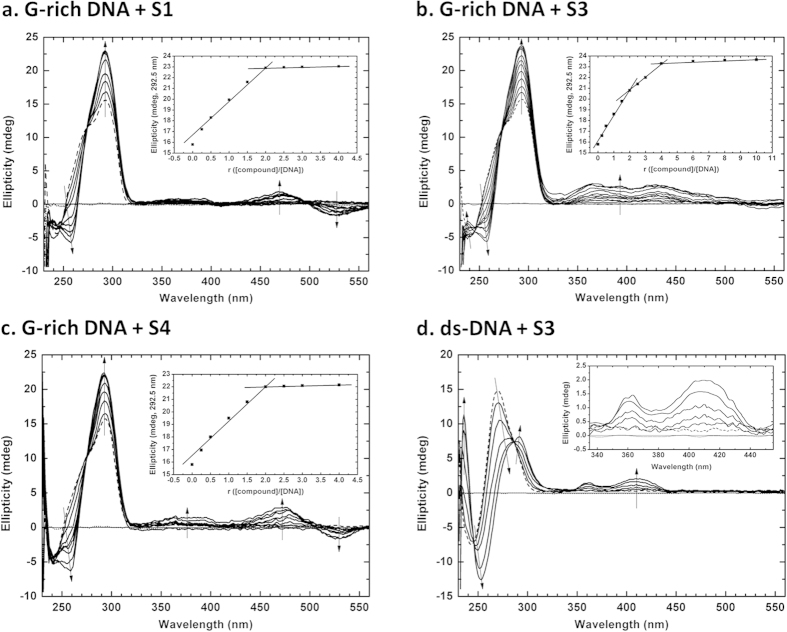
CD spectra for the interaction between benzothioxanthene derivatives S1, S3, S4 and 26 nt telomeric G-rich DNA sequences of human chromosome in 10 mM K^+^ by titration of S1, S3 and S4 into DNA. All experiments were performed in a buffer (pH 7.4) containing 10 mM Tris–HCl, 10 mM KCl, 0.1 mM EDTA, and samples of 4 μM G-rich DNA (in single strand) and 2 μM double-stranded DNA (in double strand) dissolved in the buffer were incubated for 24 h after annealing at 95 °C. (**a**) The CD spectrum for G-rich-DNA in the presence/absence of S1 at the ratio of S1 to DNA (r), 0.0, 0.25, 0.5, 1.0, 1.5, 2.0, 2.5, 3.0, 3.5, 4.0, of which the inset is the change of CD with the r ([compounnd]/[DNA]) at 292.5 nm. (**b**) The CD spectrum for G-rich-DNA in the presence/absence of S3 at the ratio of S3 to DNA (r), 0.0, 0.25, 0.5, 1.0, 1.5, 2.0, 2.5, 3.0, 4.0, 6.0, 8.0, 10, of which the inset is the change of CD with the r at 292.5 nm. (**c**) The CD spectrum for G-rich-DNA in the presence/absence of S4 at the ratio of S4 to DNA (r), 0.0, 0.25, 0.5, 1.0, 1.5, 2.0, 2.5, 3.0, 3.5, 4.0, of which the inset is the change of CD with the r at 292.5 nm. (**d**) The CD spectrum for ds-DNA in the presence/absence of S3 at the ratio of S3 to DNA (r), 0.0, 1.0, 2.0, 4.0, 8.0, 16, of which the inset is the enlarged view of ICD in the range from 355 to 455 nm. Arrows denote the increase of the compound concentration. Dashed line: DNA alone; dotted line: compound alone.

As shown in Fig. 3a–c, CD spectra of the G-rich DNA alone (dashed line) presented a strong positive band at 292.5 nm, a shoulder band around 275 nm and a relatively shallow negative band at 243 nm, which is characteristic of a hybrid type of G-quadruplex, also named a 3 + 1 G-quadruplex structure^33^, possibly including a small amount of antiparallel one which is in good agreement with EMSA showing in Fig 1; whereas three compounds alone all are non optically active there existing not any CD bands (dotted line). More importantly, CD spectrum of the G-rich DNA exhibited great difference in peak intensity, the feature of induced CD (ICD) and stoichiometry of compound-DNA interaction in the presence of **S1**, **S3** and **S4**, respectively. In Fig. 3a, with gradual titration of **S1** into the G-rich DNA at the ratios of compound/DNA, 0.25:1, 0.5:1, 1:1, 1.5:1, 2.0:1, 2.5:1, 3.0:1 and 4.0:1, the 292.5 nm positive peak intensity of the G-rich DNA CD spectrum progressively increased up to saturation at 2:1, and gradually increasing and shifting to 260 nm negative bands and 240 nm positive bands, characteristic of an antiparallel G-quadruplex structure^36^, appeared, with induced CD (ICD) spectra ranging from 560 nm to 425 nm and from 410 nm to 335 nm. Furthermore, The CD profiles have two isoelliptic points at 275 nm and 244 nm, indicating the two-state nature of the structural transition between the quadruplexes upon **S1** binding. In addition, in the inset of Fig. 3a, that the positive CD signals at 292.5 nm were plotted against compound–DNA ratio indicates stoichiometry of a 2:1 **S1**-DNA complex, presumably their interaction mode being by stacking of **S1** on two terminal G-tetrads of the G-quadruplex^37^. These results suggest that the G-quadruplex structures formed by the G-rich DNA alone gradually convert to ones dominatingly including an antiparallel one in the presence of **S1**, consistent with gel electrophoresis results shown in Fig. 1, and there exists stronger binding of **S1** to the antiparallel G-quadruplex at the ratio of 2:1 than to the 3 + 1 one. Likewise, the CD spectra of G-rich DNA in the presence of **S4** were obtained (Fig. 3c), showing that there was almost the same characteristics of the CD spectra as ones observed in the presence of **S1**, with exception of slightly strong ICD signals ranging from 555 nm to 425 nm and from 410 nm to 335 nm. However, very striking, as shown in Fig. 3b, were several quite differences in binding affinity, ICD, binding ratio of **S3** to the G-rich DNA in comparison with **S1** and **S4**. With increasing concentration of **S3**, the positive peak intensity at 292.5 nm much increase up to saturation at the ratio of **S3** to DNA 4:1 and a 240 nm distinct band and stronger ICD spectrum bands ranging from 500 nm to 435 nm appeared; the inset in Fig. 3B of the CD signal intensity at 292.5 nm as a function of **S3**/DNA (r = 0, 0.25, 0.5, 1.0, 1.5, 2.0, 2.5, 3.0, 4.0, 6.0, 8.0, 10) indicated that there were two binding modes of **S3** to the antiparallel G-quadruplex formed through inducing the 3 + 1 structure at the ratios of 2:1 and 4:1, respectively. In contrast, **S1**, **S3** and **S4** have no influence on C-rich DNA as a reference, a complementary strand of 26 nt telomeric G-rich DNA, at tested compound concentrations of 4, 20, 40 μM, which can be assigned to a single strand DNA structure with a positive maximum at 271 nm and a negative minimum at 248 nm (Fig. S4A, B and C)^38^,^39^.

**Figure 4:**
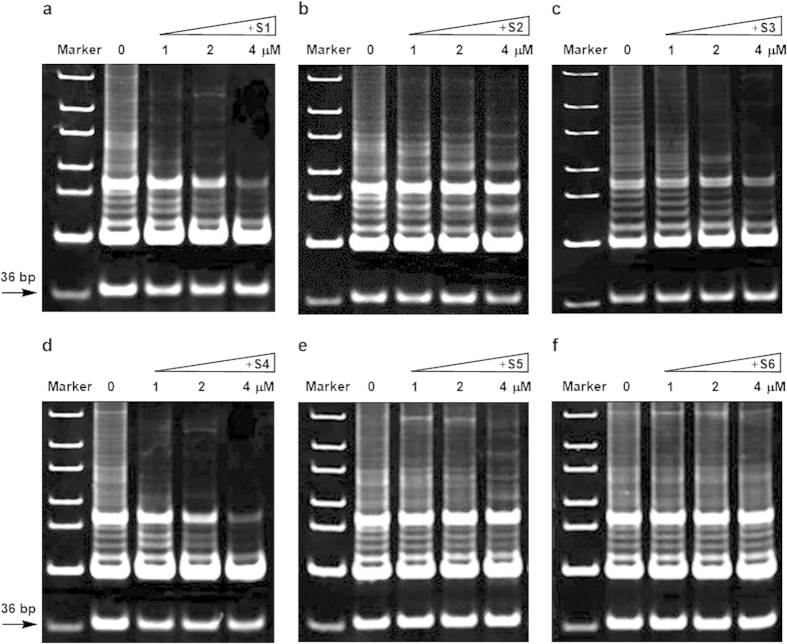
Effect of benzothioxanthene derivatives on the telomerase activity in A549 cells. Cell extracts containing telomerase and TRAP assay reagents were mixed and the activity of telomerase was determined in the presence of 0, 1, 2, or 4 μM **S1**–**S6** (**a**–**f**). The arrow indicates the 36 bp internal control.

To investigate the selectivity of benzothioxanthene derivatives binding G-quadruplex vs double stranded DNA, we have measured CD spectra of the 26 nt telomeric double-stranded DNA (ds-DNA, Table 1), in the presence and absence of representative **S1**, **S3** and **S4**. As seen in Fig. 3d, the CD spectrum of the ds-DNA alone was characteristic of double stranded DNA structure with strong positive and negative bands at 270 nm and at 243 nm, respectively^40^,^41^. Whereas gradual titration of **S3** into the ds-DNA at increasing concentrations of 2, 4, 8, 16, 32 μM resulted in more dramatic spectral changes in shape, ICD feature, that an original positive peak at 270 progressively decreased, meanwhile red-shifted, and then increased to give rise to a clear positive band at 292 nm, and that a original negative band at 243 nm progressively increased, meanwhile red-shifted to produce a more pronounced negative bands at 254 nm, accompanied by a new strong positive peak at 236 nm presenting, which were characteristic of the antiparallel G-quadruplex, consistent with the conversion results of the G-rich DNA CD spectra in Fig. 3a–c. Meanwhile, two isoelliptic points near 286 nm, 245 nm and ICD spectra enlarged in the inset of Fig. 4d ranging from 445 nm to 345 nm in line with the ICD showing in Fig. 4b were observed. These observations hint that ds-DNA gradually converts to a mixture of several conformers mainly including an antiparallel G-quadruplex in agreement with one shown in the G-rich DNA CD spectra in Fig. 4b, in the presence of **S3**. In contrast, **S1** and **S4** had relatively a little effect on ds-DNA CD spectra (Figs S4G and H).

In addition, we obtained CD spectra of Mut-DNA (Figs S4D, E and F) as a negative control in the presence and absence of **S1**, **S3** and **S4**, respectively, under the same experimental conditions as above used ones, which can not form any G-quadruplex structure due to the substitution of cytosine base for the middle guanine base of each of four three-Guanine-runs in G-rich DNA (Table 1), showing that Mut-DNA alone was typical of a freedom single-stranded DNA structure with a positive band at 275 nm and a negative band at 249 nm, and no influence was observed in the presence of compounds.

To sum up, CD results suggest that examined compounds **S1**, **S3** and **S4** can induce the formation of and stabilize an antiparallel G-quadruplex structure formed in 26 nt telomeric G-rich and double-stranded DNAs, and possess stronger affinity and higher specificity for the antiparallel G-quadruplex over C-rich DNA, Mut-DNA and ds-DNA, of which **S3** is a strongest inducer and stabilizer for such an antiparallel G-quadruplex.

### Real-time monitoring apoptosis of cancer cell lines induced by S1–S6

To evaluate the cytotoxicity and kinetics of cytotoxicity of six benzothioxanthene derivatives **S1**–**S6** to two types of cancer cell lines, a real-time cell analysis (RTCA) technique was used to dynamically monitor changes of cells, including cell number, kinetics behavior of cell apoptosis and so on, exposed to 0, 0.25, 0.5, 1.0, 2.0 or 4.0 μM examined compounds. The RTCA technique measures changes in the impedance of individual microelectronic wells that is correlated linearly with changes in cell numbers in log phase of cell growth, thus allowing determination of cytotoxicity. These impedance measurements are expressed as a cell index (CI) value, which corresponds to cell number, shape, and degree of substrate attachment^42^. A chemically induced decrease in the CI indicates that fewer cells are interacting with the microelectrodes due to diminished viability or disruption of the cellular footprint; while an increased CI can reflect higher cell numbers, increased cell adhesion or changes in cell morphology (e.g. increased cell/electrode contact area due to cell spreading)^43^,^44^. Also, based on experimental curves (CI *vs* time), IC_50_ of drugs inhibiting cell lines can be calculated by RTCA system software. Therefore, this methodology can provide some useful analyses for various viable cell activities, drug-induced cellular apoptosis, and kinetics of cytotoxicity responses for drugs.

As shown in Figs S5 and S6, cell culture medium alone as a negative control and 0.5% DMSO-only as a reference almost had no effect on viability of tested cancer cells. Fig. S5 and Fig. S6 showed cell indexes of A549 and SGC7901 with time, respectively, in the presence and absence of different concentrations of **S1**–**S6**, with IC_50_ values of drugs inhibiting two cancer cell lines derived from CI curves (Table 3). We found the fact that viability of both cells remarkably lowered and the time required to induce the cell apoptosis decreased with increasing concentrations of compounds, but, very interestingly, those slightly did with increasing **S6** amount in spite of great similarity in structure to other used compounds **S1**–**S5** (Figs S5 and S6); in any case, however, the apoptosis of cells was dose-dependent. As seen in Figs S5 and S6, exposure to **S1**, **S3** and **S4** produced quickly decreases in the CI of both cells, of which **S3** decreased most quickly the CI values at selected concentrations, even though it almost induced the apoptosis of all cancer cells at a lower concentration of 2 μM (Table 3). Those findings are in good agreement with above experimental outcomes by EMSA, UV melting and CD assays.

In addition, it is noteworthy that different cytotoxicity response patterns were also seen between two cell types and among compounds. The time-course of CI changes in A549-seeded microwells was gradual with the increasing **S3** concentrations; whereas the time-course of CI changes in SGC7901-seeded microwells rapid at the first three concentrations of 0.25, 0.5, 1.0 μM. In the case of **S5**, the time-course of CI changes in SGC7901-seeded microwells was similar to those in A549-seeded microwells; whereas the time-course of CI changes in A549-seeded microwells rapid at the final one concentration of 4.0 μM. Furthermore, for an identical type of cell line there were different cytotoxicity response patterns among compounds in RTCA profiles shown in Figs S5 and S6. These observations may be because there exist different kinetics mechanisms of apoptosis of two cancer cell lines in the presence of different compounds. In all, RTCA profiling reveals that **S1**, **S3** and **S4** possess stronger *in vitro* cytotoxicity for both of tested cancer cells and for SGC7901 cells than that for A549 cells, in particular, **S3** with IC_50_ values of 0.53 μM for the former cells and 0.60 μM for the latter, and their differentiated apoptosis responses possibly originate from different kinetics modes of exposed cells to compounds.

### In-vitro inhibition of telomerase activity by benzothioxanthene derivatives S1–S6

**Figure 5:**
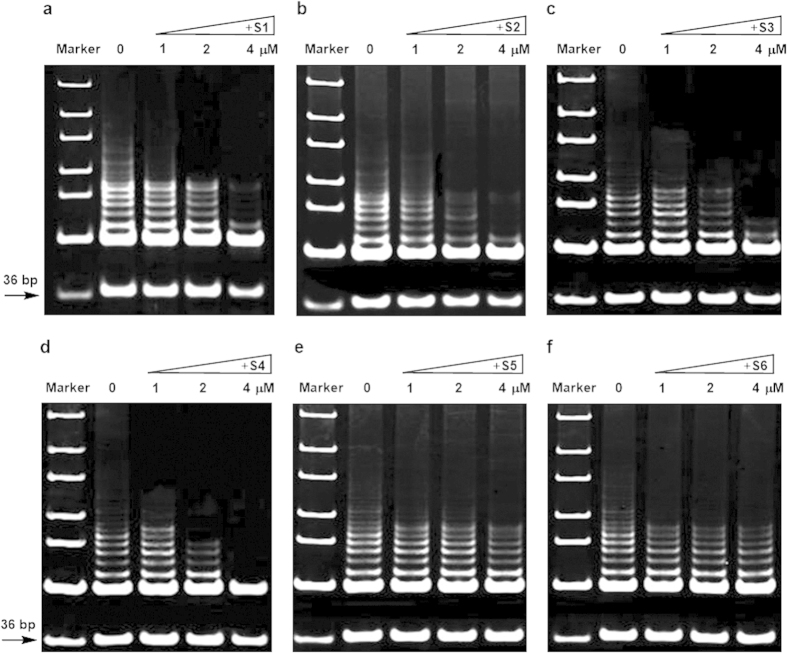
Effect of benzothioxanthene derivatives on the telomerase activity in SGC7901 cells. Cell extracts containing telomerase and TRAP assay reagents were mixed and the activity of telomerase was determined in the presence of 0, 1, 2, or 4 μM **S1**–**S6** (**a**–**f**). The arrow indicates the 36 bp internal control.

The effect of benzothioxanthene derivatives on telomerase activity in A549 cells and SGC7901 cells, both of which express telomerase, was examined using telomerase repeat amplification protocol (TRAP) assay. As shown in Figs 4 and 5, a telomeric DNA ladder was observed in the absence of the compounds, whereas **S1**, **S3,** and **S4** significantly inhibited the formation of the ladder in a compound concentration-dependent manner, ranging from 1 μM to 4 μM. By contrast, the effect of compounds **S2**, **S5**, and **S6** on the telomerase activity was marginal. In addition, it seems that compounds presented stronger telomerase activity to SGC7901 than to A549. These data are consistent with the above experimental results.

### Effect of benzothioxanthene derivatives on the mobility of tumor cells

**Figure 6:**
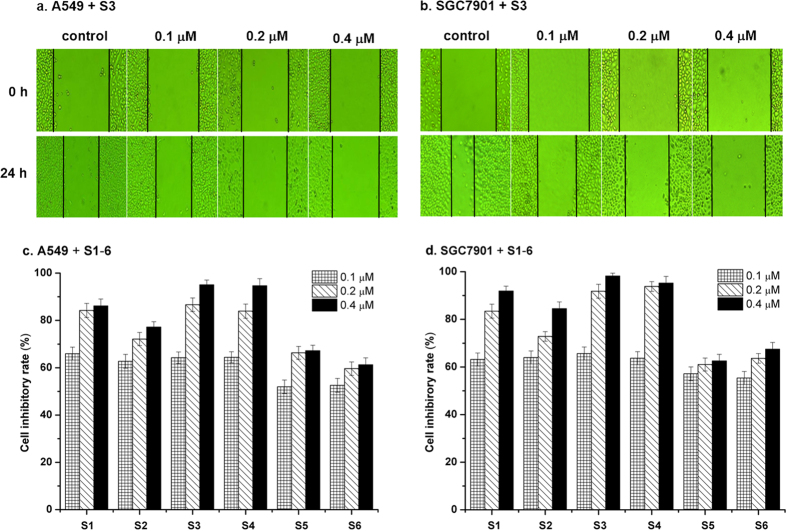
Effect of benzothioxanthene derivatives on the mobility of tumor cells. Cell wound healing assay was conducted in A549 (**a**) and SGC7901 (**b**) cells which were grown in culture dish and streaked with a pipet chip. After the addition of **S3** to the culture at the indicated concentrations for 24 h, mobility of the cells was recorded under a microscope. In the bar charts, cell inhibitory rate (%) for suppression of the migration in A549 (**c**) cells and SGC7901 (**d**) cells as a function of concentrations of compounds **S1–S6** showed mean results of three independent experiments in triplicates, where the width of the streaked line was measured at four to six reference points along the originally streaked line. The % cell inhibitory rate was calculated by the equation: cell inhibitory rate (%) = (1-D_drug_/D_control_) × 100%, where D_drug_ is mean distance of cell migration in drug group, D_control_ is mean distance of cell migration in control group; The % cell inhibitory rate for control is zero. Values are the means ± S.D. (P < 0.05). Other would healing assays are presented in Figure S7.

Cell mobility plays an essential role in tumor metastasis and prognosis of cancer. We thus assessed the effect of **S1**–**S6** on the migration of two species of cancer cells in an *in-vitro* wound healing model. When tumor cell culture was streaked with a pipet tip, an empty space was created, which was filled by the tumor cells by a combination of proliferation and cell migration as shown in Fig. 6 and Fig. S7. The addition of benzothioxanthene derivatives to the cell culture, however, significantly reduced the number of cells in the empty space. As shown in Fig. 6c,d, cell inhibitory rates for two types of cancer cells are greater than 60% after 24 h treatment with all compounds, in particular, **S1**, **S3** and **S4**, at the concentration of 0.2 μM; furthermore, all compounds gave rise to higher cell inhibitory rates for SGC7901 than those for A549 at the concentration of 0.4 μM, but **S5**. Meanwhile, we observed that compound **S3** was strongest inhibitor to the cell migration with inhibitory rates of 94.1% (±1.8) for A549 cells and 97.2% (±1.6) for SGC-7901 cells. These data are in good agreement with foregoing experimental results and clearly demonstrate that compounds **S1**–**S6** suppressed the mobility of tumor cells.

## Discussion

Telomeres are located at the ends of human chromosomes and play an important role in the protection of chromosomes from degradation and unwanted recombination and end-to-end fusion, as well as the determination of the number of cell division^2^,^5^. It is well established that telomeres in somatic cells are gradually shortened as a consequence of the end-replication effect due to the lack of Okazaki fragments, leading to chromosome instability and subsequent cell apoptosis or senescence^45^,^46^. The terminal 100–250 nucleotides at the 3′ end of telomeric nucleotides are single-stranded, but protected by POT1, a single-stranded DNA binding protein, which regulates the activity of telomerase. Telomerase elongates the telomere and maintains the telomere-length homeostasis in cells that need to divide regularly, such as male germ cells, activated lymphocytes, and certain adult stem cells, whereas other somatic cells do not express it^5^,^7^. It is worthy of note that more than 85% of cancer cells and primary tumors express telomerase, which confers tumor cells infinite proliferation capacity. Telomerase is, therefore, a promising target to develop therapeutics for various types of cancer^7^,^13^,^14^,^47^. Because the activity of telomerase is modulated by POT1 and because the recruitment of hPOT1 to the single-stranded telomeric DNA is hampered by the formation of G-quadruplex structure at the single-stranded nucleotides, it is reasonable to hypothesize that the inducer or stabilizer of G-quadruplex complexes can be a promising candidate for therapeutics for cancer. In addition, G-quadruplex sequence is widely prevalent in the genome of a eukaryotic telomere, especially gene promoter and 5′-UTR sequences involved in cellular proliferation. More recently, more encouraging is that the existence and persistence of G-quadruplex structures has been confirmed existing in the genomic DNAs of human cells^48^,^49^. It is thus likely that small compounds which promote or stabilize the formation of G-quadruplexes inhibit the telomerase activity as well as the proliferation of tumor cells.

It has been reported that certain small molecules could stabilize G-quadruplex complexes and inhibit maintenance, transcription, and translation of telomere^16^,^50^,^51^. Based on the findings, many laboratories have explored small compounds that could promote the formation and stabilization of G-quadruplex complexes and exhibit beneficial effects in the treatment of cancer. In 1997, Hurley and his colleagues for the first time discovered and demonstrated that a small molecule, 2,6-diamidoanthraquinone derivative could stabilize the G-quadruplex structure and inhibit the activity of telomerase^52^. Quarfloxin is the first therapeutic agent designed to target G-quadruplex, and was evaluated clinically in the treatment of carcinoid or neuroendocrine tumors in early phase II trials^53^.

We previously synthesized benzoxanthene derivatives with 1,8-naphthalimide backbone structure that could bind to G-rich telomere nucleotides, inhibit human telomerase, and exhibit potent tumoricidal activity. The compounds, however, present poor hydrophilicity and almost failed to show substrate specificity and interacted with both G-quadruplexes and double-stranded nucleotides, possibly leading to unexpected adverse reactions via binding to critical promoters and/or enhancers. In the present study, we designed and reported six novel benzothioxnathene derivatives **S1**–**S6** with protonated side chains, which exhibited increased solubility in water and anti-tumor activity, and promoted the antiparallel G-quadruplex formation. Our work has confirmed that the benzothioxanthene derivatives, especially **S1**, **S3** and **S4**, possessed the high selectivity and excellently inducing the formation and stabilizing capability for the G-quadruplex DNA rather than for double-stranded DNA, C-rich single-stranded DNA and Mutated single-stranded DNA based on EMSA, UV-melting and CD studies, but **S3** also can interact with 26 nt telomeric double-stranded DNA which was induced to partly form the antiparallel G-quadruplex (Fig. 3d), and also displayed cumulative cytotoxic, anti-proliferative and cell migrating inhibition effects in A549 cells and SGC7901 cells based on RTCA, trap assay and wound healing studies. Maybe such selectively binding feature was because the side chains with positive charges and the overall large steric space of the compounds can help bind to the antiparallel G-quadruplex by an end-stacking mode and prevent the interaction with other tested DNAs, but more details should be further explored. The observation indicates that the novel benzothioxanthene derivatives specifically bound to the telomeric G-rich DNA and might interfere with the recruitment of POT1, resulting in the failure in the recruitment and/or activation of telomerase. The present TRAP assays clearly demonstrated that the derivatives, especially **S1**, **S3**, and **S4**, inhibited the catalytic activity of telomerase in a concentration-dependent manner. The findings strongly suggest that newly synthesized benzothioxanthene derivatives with protonated side chains inhibit tumor growth and exhibit anti-tumor activity through specifically binding to the telomeric antiparallel G-quadruplex structure and subsequent down-modulation of telomerase. Also, this is greatly possibly a molecular mechanism for biological activities found of examined compounds interacting with two species of cancer cells. But further studies will investigate precise mechanisms for the differential kinectic response in cytotoxicity for an identical species of cancer cells exposed to different compounds and for different species of cancer cells exposed to an identical compound, for telomerase inhibition and for the migration of tumor cells observed.

Anti-tumor effects include the inhibition of tumor growth and metastasis. The present assay using wound healing model showed that the benzothioxanthene derivatives inhibited the wound healing *in vitro*. Because wound healing is achieved by active proliferation and migration of cells, the results indicate that the compounds inhibited proliferation and/or migration of tumor cells. Although the present compounds exhibit anti-tumor activity only at relatively moderate concentrations ranging from 0.1 μM to 4 μM, further optimization structure of this group of compounds would provide more preferable and clinically beneficial therapeutics in the treatment of cancer.

In addition, more noteworthy are that, based on our experiments *in vitro* and in a DNA molecular level, **S3** exhibited the most excellent effects in affinity, specificity, cytotoxicity, inhibition of telomerase and migration for tested targets including DNAs and cancer cells among examined compounds, and that **S3** binding to the G-quadruplex possibly finally adopted a 4:1 interaction mode, in which two molecules are by end-stacking at a 2:1 ratio on both of the terminal G-tetrads of the G-quadruplex, and the other two molecules by binding to the grooves of the G-quadruplex (Fig. 3b). The two hydroxyl groups in the side chain of **S3** that can form additional hydrogen bonds with DNA bases may sufficiently account for the more efficient targets binding results for **S3** 21,22, in addition to the basic interaction natures of this type of compounds and their targets, including the side-chain directing to DNA grooves followed by positively charged nitrogen binding to the negative phosphorate backbone and two molecules end-stacking at a 2:1 ratio on both of the terminal G-tetrads of the G-quadruplex, but this is possibly not conclusive.

In conclusion, the newly synthesized benzothioxanthene derivatives containing protonated side chains, especially **S1**, **S3**, and **S4**, specifically promoted the formation and stabilization of the antiparallel G-quadruplex formed by 26 nt telomeric G-rich nucleotides. Furthermore, the compounds could inhibit telomerase activity, interfere with cell proliferation and migration, and induce tumor cell apoptosis. Of those **S3** is more worthy of attention due to giving rise to the strongest interacting effects on targets. Our results indicate that the novel derivatives exert anti-tumor activity through the specific interaction with telomeric G-quadruplex structures and the subsequent inhibition of telomerase. Although much needs to be done including systematic structure optimization of novel benzothioxanthene derivatives, this work encourages us to further investigate the precise mechanism underlying the small molecule-mediated inhibition of telomerase and the differential cytotoxicities, and to explore new telomerase inhibitors including benzothioxanthene derivatives.

## Methods

### Materials

Target compounds **S1**–**S6** were synthesized according to the established method by our Lab in Figure S1 (see detailed synthetic procedure and characterization of compounds in Supplementary Fig. S1 and synthetic section). In brief, firstly, *ortho*-aminobenzenethiol (1.13 equiv) was added dropwise into a solution of 4-bromo-1,8-naphthalic anhydride **S7** in DMF (3.33 mL/mmol) in the presence of K_2_CO_3_ (0.5 equiv) and the resulting mixture was stirred under N_2_ atomsphere at reflux for 2 h till starting material **S7** was reacted completely by TLC. Then the mixture was cooled, put into water and filtered to give 4-(2-aminobenzenethio)-1,8-naphthalic anhydride **S8** as dark green solid without by-product formation, yield 94.0% (mp 199–201 °C, lit.^54^ 200–201 °C). Secondly, a solution of sodium nitrate (10 equiv) in water was added dropwise slowly to a solution of the above intermediate in acetic acid (3.21 mL/mmol) during 20 min at 0–5 °C and stirred for additional 8 h. Then, the reaction mixture was added to a solution of CuSO_4_ (4.43 equiv) in water and stirred at boiling temperature for 2 h. After solvent was removed, the residual was separated by silica gel chromatography to give benzothioxanthene-3,4-dicarboxylic anhydride **S9** as pure orange solid, yield 73.0% (mp >300 °C, lit.^55^ 321–324 °C). Thirdly, an excess of diamine (1.5 equiv) was added into a suspension of **S9** in ethanol and the resulting solution stirred at reflux for 4 h. After removal of solvent, the residual was isolated by silica column chromatography (CH_2_Cl_2_: MeOH = 8:1, v/v) to afford benzo[*k*, *l*]thioxanthene-3,4-dicarboximide derivatives **S1a**–**S6a**, as a yellow red solid, yield over 92%. Finally, **S1a**–**S6a** were dissolved in methylene chloride and through the solution dry hydrogen chloride passed at room temperature for 1.5 h. After removal of solvent, the residual was purified on silica gel chromatography (CH_2_Cl_2_: MeOH = 12:1, v/v) to give final desired compounds **S1**–**S6** as a yellow solid, yield 90–93%.

All compounds were dissolved in DMSO to give a concentration of 10 mM and stored as stock solution. DNA oligomers/primers were purchased from Sangon (Shanghai, China), and used without further purification. These oligomers were purified by HPLC, identified by HPLC-CE and TOF Mass, and exhibited single-band electrophoretic mobilities in denaturing polyacrylamide gel electrophoresis with stated purities of ≥95%. The sequences of DNA oligomers used are listed in Table 1. Single-stranded DNA concentrations were determined by measuring the absorbance at 260 nm at high temperature. The different DNA strands were mixed in equimolar amounts and the total species concentrations were estimated by averaging the extinction coefficients of the single strands^55^. Solutions of the DNA oligomers were prepared as follows: an oligonucleotide sample dissolved in a buffer containing 10 mM Tris–HCl, 10 mM KCl, 1 mM EDTA (pH = 7.4) was heated at 95 ^o^C for 5 min and allowed to slowly cool to room temperature at 1 °C/1 min over a period of several hours and then incubated at 4 ^o^C overnight. A549 (Human lung adenocarcinoma cell line) and SGC7901 (Human stomach cancer cell line) were supplied by China General Microbiological Culture Collection Center (CGMCC, Beijing, China). All other commercially available reagents and solvents were purchased and used without further purification unless otherwise stated. MilliQ water was used to prepare buffer solutions.

### Electrophoretic mobility shift assay

The electrophoretic mobility shift assay (EMSA) was conducted using 20% native polyacrylamide gel electrophoresis and 1× TBE (Tris base-boric acid-EDTA) buffer solution. The gels were run at 150 V for 4 h in 4 ^o^C circulating cooling water. The gels were then immersed in 1× SYBR Gold and in 1× TE solution for 30 min, respectively, rinsed with ultrapure water, and then photographed using a Bio-Rad gel imaging analyzer.

### UV melting assay

Temperature-dependent absorption was measured using a UV-2550 spectrophotometer (Shimadzu) equipped with a thermoelectrically controlled cell holder and quartz cells with a path length of 10 mm. The absorbance at 295 nm for 2 μM single-stranded DNAs, including G-rich DNA, C-rich DNA and Mut-DNA, and at 260 nm for 2 μM double-stranded DNA (ds-DNA) in a buffer (pH 7.4) containing 10 mM Tris–HCl, 10 mM KCl, 0.1 mM EDTA, in the presence and absence of compounds and the buffer as a reference was monitored with the temperature being ramped between 1 ^o^C and 95^°^C at 1.0 ^o^C/min and the samples being allowed to equilibrate for 10 min at each temperature setting. The increasing concentrations of compounds were used at 0, 4, 8, 16 μM for absorbance measurements of G-rich DNA and at 0, 16 μM for those of C-rich DNA, Mut-DNA and ds-DNA. Dry nitrogen was passed through the sample chamber to prevent condensation.

### Circular dichroism (CD) assay

The CD experiments were performed using a Jasco-815 CD spectropolarimeter. Spectra were baseline-corrected and the signal contributions of the buffer were subtracted. Each titration data was obtained from 560 to 220 nm with a 5 mm path length quartz cuvette at 25 °C, averaged from at least three successive accumulations at a scan rate of 100 nm/min using a bandwidth of 5.0 nm at a standard sensitivity. At first, DNA solutions and compound solutions alone, respectively, were scanned under the determined conditions. And each compound was then titrated into the same cuvette at the selected increasing concentration ratios, and the obtained complexes equilibrated after each titration for 15 min prior to scan, and finally scanned under the equal conditions.

### Cell culture

Cells were routinely maintained in RPMI 1640 (Invitrogen Gibco) supplemented with 10% FBS (Invitrogen Gibco) and penicillin-streptomycine (Sigma-Aldrich, 100 U/mL) at 37.0 °C in a humidified atmosphere containing 5% CO_2_.

### Cytotoxicity assay

Cytotoxicity assay was performed by using a real time cell analyzer (RTCA) system comprising modified 96-well plates with each well consisting of a top chamber and a bottom chamber separated by a microporous membrane. Initially, 95 μL of cell culture medium was added to the lower and upper chambers and the 96 E-plate was locked in the RTCA DP device at 37 °C with 5% CO_2_ for 30 min to equilibrate temperature according to the manufacturer’s instructions. Then, 100 μL of each tumor cell suspensions were dispensed into 96-well E-plates at a final concentration of 8000 cells per well. The growth status of the tumor cells was recorded through an RTCA system until the cells reached to logarithmic growth phase (approximately 24 h after seeding). Plates were then removed from the incubator. Each well was treated with a 5 μL of compound solution according to the designed concentration, DMSO solution in RPMI 1640 at a final concentration of 0.5% as a reference and RPMI 1640 as a negative control. The treatment layout was randomized for each replicate to avoid layout-related artifacts. After treatment, plates were returned to the RTCA unit in the incubator and monitored hourly until a growth plateau had been reached, usually ~60 h post-treatment. Three independent replicate experiments were performed by seeding triplicate microwells per treatment group.

### Telomerase activity assay

Telomerase activity of A549 cells and SGC7901 cells was detected by TRAPEZE® Telomerase Detection Kit (S7700-KIT; Millipore Company, Purchase, NY) according to the manufacturer’s instructions and the literature^56^. In brief, cell pellet was resuspended in 200 μL of 1× CHAPS lysis buffer/10^5^–10^6^ cells. Next, positive control cell pellet provided in the kit was resuspended with 200 μL of 1× CHAPS lysis buffer, and the suspension was incubated on ice for 30 minutes. Then, 160 μL of the supernatant was transferred into a fresh tube, and protein concentration was determined. The remaining extract was aliquoted, quick-frozen on dry ice, and stored at −85 °C to −75 °C. After determination of protein concentration, 10–750 ng/μL protein in cell extract was incubated with TRAP buffer (20 mM Tris–HCl, pH 8.3, containing 1.5 mM MgCl_2_, 63 mM KCl, 0.05% (v/v) Tween 20, 1 mM EDTA, and 0.01% BSA; TRAPeze telomerase detection kit), supplemented with dNTP mix, TS primer, TRAP primer mix, dH_2_O, Taq polymerase and compounds at indicated concentrations at 30 ^o^C for 30 min, 94 ^o^C for 4 min and PCR was performed at 94 ^o^C for 30 s, 59 ^o^C for 30 s , 72 ^o^C for 1 min for 30 cycles and 72 ^o^C for 7 min in a thermocycler (Applied Biosystems Verity Thermal Cycler; ABI). PCR samples were run on a 10% (w/v) native-PAGE gel in 0.5× TBE for 4 h at 100 V. After electrophoresis, the gel was stained with SYBR GOLD and was destained for 30 min at room temperature.

### Wound healing assay

A549 and SGC7901 cells were grown in DMEM medium containing growth factors at the cell density of 1 × 10^5^ cells/mL for 24 h. A disposable 200 μL plastic pipette tip was used to scratch the monolayer of cells in a streaking motion. Compounds were added to the streaked cell culture at the indicated concentrations. The streaked cells were then cultured in serum-free medium for an additional 24 h and photographed. To quantify the experimental results, the % cell inhibitory rate was calculated by the equation: cell inhibitory rate (%) = (1-D_drug_/D_control_) × 100%^57^, where D_drug_ is mean distance of cell migration in drug group, D_control_ is mean distance of cell migration in control group, with pictures of the initial wounded monolayers being compared with the corresponding pictures of cells at the end of the incubation, and data are presented as mean ±SD. Artificial lines fitting the cutting edges were drawn on pictures of the original wounds and on the pictures of cultures after incubation. Three different points were marked on each plate. Representative images of three independent experiments were shown.

### Statistical analysis

The statistical significance of experimental results was evaluated using unpaired Student’s t-tests or one-way ANOVA analysis. Data were expressed as means ± standard deviations (S.D.) of three independent experiments and the difference (P < 0.05) was considered significant.

## Additional Information

**How to cite this article**: Zhang, W. *et al.* Formation and stabilization of the telomeric antiparallel G-quadruplex and inhibition of telomerase by novel benzothioxanthene derivatives with anti-tumor activity. *Sci. Rep.* **5**, 13693; doi: 10.1038/srep13693 (2015).

## Supplementary Material

Supplementary Information

## Figures and Tables

**Table 1:** The sequences of DNA oligomers used in this study.

Oligomer	Sequence
Tel26nt-G-rich-strand DNA (G-rich DNA)	5′-ttaGGGttaGGGttaGGGttaGGGtt-3′
Tel26nt-C-rich-strand DNA (C-rich DNA)	5′-aaCCCtaaCCCtaaCCCtaaCCCtaa-3′
Tel26nt-Mutated-strand DNA (Mut-DNA)	5′-ttaGCGttaGCGttaGCGttaGCGtt-3′
Tel26nt-double-stranded DNA (ds-DNA)	5′-ttaGGGttaGGGttaGGGttaGGGtt-3′
	3′-aatCCCaatCCCaatCCCaatCCCaa-5′

**Table 2:** ΔT_m_ (°C) values for G-rich DNA interaction with benzothioxanthene derivatives **S1–S6.**

G-rich-DNA/compound	ΔT_m_ (°C)^a^					
	S1	S2	S3	S4	S5	S6
1:2	15.1 ± 0.3	14.5 ± 0.2	15.4 ± 0.4	20.5 ± 0.3	13.2 ± 0.2	9.2 ± 0.1
1:4	17.4 ± 0.2	17.1 ± 0.3	27.0 ± 0.3	23.4 ± 0.3	15.3 ± 0.3	11.5 ± 0.2
1:8	19.8 ± 0.4	19.0 ± 0.3	29.8 ± 0.5	25.6 ± 0.6	15.7 ± 0.2	12.1 ± 0.2

^a^Standard deviation is given.

**Table 3:** IC_50_ of **S1**–**S6** inhibiting two tumor cell lines (μM).

Cell	S1	S2	S3	S4	S5	S6
A549	0.78 ± 0.07^a^	1.54 ± 0.12	0.60 ± 0.04	1.13 ± 0.08	3.21±0.12	>>4
SGC7901	0.70 ± 0.09	1.10 ± 0.10	0.53 ± 0.05	0.87 ± 0.06	1.62 ± 0.11	>4

^a^Standard deviation is given. IC_50_ value: Drug concentration producing 50% cancer cell death.
