# Supplementary material for: Formation and stabilization of the telomeric antiparallel G-quadruplex and inhibition of telomerase by novel benzothioxanthene derivatives with anti-tumor activity
Source: Sci Rep. 2015 Sep 2;5:13693. doi: 10.1038/srep13693 (PMC4557076; doi:10.1038/srep13693)
Supplement: Supplementary Information [file srep13693-s1.doc]

**Supplementary information**

**Formation and stabilization of the telomeric antiparallel G-quadruplex and inhibition of telomerase by** **novel benzothioxanthene derivatives with anti-tumor activity**

Wen Zhang^1^*, Min Chen^1^, Yan Ling Wu^2^*, Yoshimasa Tanaka^3^, Yan Juan Ji^1^, Su Lin Zhang^1^, Chuan He Wei^1^ & Yan Xu^4^

^1^Lab of Chemical Biology and Molecular Drug Design, College of Pharmaceutical Science, Zhejiang University of Technology, 18 Chaowang Road, Hangzhou, 310014, China, ^2^Lab of Molecular Immunology, Zhejiang Provincial Center for Disease Control and Prevention, 3399 Binsheng Road, Hangzhou, 310051, China, ^3^Center for Innovation in Immunoregulative Technology and Therapeutics, Graduate School of Medicine, Kyoto University, Kyoto, 606-8501, Japan, ^4^Division of Chemistry, Department of Medical Sciences, Faculty of Medicine, University of Miyazaki, 5200 Kihara, Kiyotake, Miyazaki, 889-1692, Japan.

Synthesis and identification of six target compounds **S1-S6:**

**Figure S1.** **Synthesis of six novel benzo[k,l]thioxanthene-3,4-dicarboximides S1-S6.** Reaction conditions: (i) 2-aminobenzenethiol, K_2_CO_3_, DMF, N_2_, reflux, 2 h, 94.0% yield; (ii) NaNO_2_, H_2_O, HOAc, HCl, 0–5 ℃ 8h; (iii) CuSO_4_, HOAc, H_2_O, reflux, 2 h, 73.0% yield; (iv) R^1^(CH_2_)_3_NH_2_, ethanol, reflux, 4 h, yield over 92%; (v) HCl(g), 1–2 h, yield 90‒93%.

**General.** Six novel benzo[k,l]thioxanthene-3,4-dicarboximides **S1**-**S6** were synthesized according to the established method by our Lab in Figure S1. Melting points were taken on a XT-5A micro- melting point measure apparatus made in Shanghai and uncorrected. Reagents and solvents were purchased from standard suppliers and used without further purification. NMR spectra were recorded with AVANCE III 500 MHz nuclear magnetic resonance spectrometer (Bruker, Switzerland). Chemical shift (δ) were reported in ppm and coupling constants (J) in herts. The following abbreviations were used to explain multiplicities: s, singlet; d, doublet; t, triplet; q, quartet; m, multiplet. ESI-TOF-Mass spectrometry was produced on an Agilent 6210 TOF LC/MS spectrometer (Agilent, America). Elemental composition was analysized on a CarloErba1106 Analyser.

(a) 4-(2-Amino-thiophenyl)-1,8-naphthalic anhydride **S8**^1^.

4.75 mL of *ortho*-aminobenzenethiol (16.96 mmol) was added dropwise into a mixture of 4-bromo-1,8-naphthalic anhydride (**S7**) (4.16 g, 15.02 mmol) and K_2_CO_3_ (1.04 g, 7.54 mmol) was mixed in 50 mL of DMF. The obtained mixture was stirred under N_2_ atomsphere at reflux for 2 h till starting material **S7** was reacted completely by TLC check. Then the mixture was cooled to room temperature and put into water (100 mL), filtered to afford dark green solid **S8** (4.34 g, 90.2% yield, mp 199.3–202.1 °C，lit.^1^ 200–201 °C).

(b) Benzothioxanthene-3,4-dicarboxylic anhydride **S9**^1^.

The above intermediate (4.34 g, 13.5 mmol) was dissolved in 140 mL of acetic acid at 0–5 °C. A solution of sodium nitrate (9.33 g, 135.31 mmol) in water (15 mL) and concentrated hydrochloric acid （42 mL）was, respectively, added dropwise slowly into the above mixture during 20 min at 0–5 °C under stirring. After that, the mixture was stirred for 8 h at room temperature. Then the reaction mixture was added to a solution of CuSO_4_ (9.58 g, 59.89 mmol) in water (170 mL) at boiling temperature and the mixture stirred for another 2 h at the same temperature. The reaction mixture was cooled to room temperature and filtered to remove solvent and separated on silica gel chromatography (CH_2_Cl_2_: n-Hexane = 6:1, v/v) to obtain pure orange solid **S9** (2.81 g, 68.4% yield, mp >300 °C, lit.^1^ >300 ℃). ^1^H NMR (500 MHz, CDCl_3_): δ 7.42-7.48 (m, 3H, 9-, 10-, 11-H), 7.57 (d, ^3^J_H,H_ = 8.05 Hz, 1H, 6-H), 8.23-8.26 (m, 2H, 5-, 8-H), 8.41 (d, ^3^J_H,H_ = 8.10 Hz, 1H, 1-H), 8.62 (d, ^3^J_H,H_ = 8.10 Hz, 1H, 2-H).

(c) General procedure for the preparation of benzo[*k*, *l*]thioxanthene-3,4-dicarboximides **S1a**‒**S6a.**

Benzothioxanthene-3,4-dicarboxylic anhydride **S9** was suspended in ethanol and an excess of diamines (1.5 equiv) was added. The mixture was stirred under N_2_ atomsphere at reflux for 4 h till **S9** was reacted completely by TLC check. After cooling, the solvent was removed by rotary evaporation and the crude product was chromatographed on a SiO_2_ column, eluted with CH_2_Cl_2_/ MeOH (8:1, v/v) to afford the proposed compound as a yellow red solid, yield over 92%.

(d) General procedure for synthesis of benzo[*k*, *l*]thioxanthene-3,4-dicarboximide hydrochlorides **S1–S6.**

Benzo[*k*, *l*]thioxanthene-3,4-dicarboximide derivatives **S1a**‒**S6a** were dissolved in methylene chloride and through the solution dry hydrogen chloride passed at room temperature for 1.5 h. After removal of solvent, the residual was purified by silica gel chromatography (CH_2_Cl_2_: MeOH = 12:1, v/v) to give final desired compounds **S1**‒**S6** as a yellow solid, yield 90‒93%.

N-(3-(dimethylamino)propyl)benzo[*k*, *l*]thioxanthene-3,4-dicarboximide hydrochloride **S1**: Benzothioxanthene-3,4-dicarboxylic anhydride **S9** was reacted with N,N-dimethyl-1,3-propanediamine according to the general procedure (c) to give the N-(3-(dimethylamino)propyl)benzothioxanthene-3,4-dicarboximide **S1a** as a yellow red solid, yield 93.0 %, mp 180.0‒182.2 °C. Then **S1a** produces **S1** in the presence of dry hydrogen chloride according to the general procedure (d) as a yellow solid. R_f_: 0.14 (DCM/methanol 12/1, v/v), yield 93.0%, mp: >300 °C. ^1^H-NMR (500 MHz, CDCl_3_): δ 2.04-2.07 (m, 2H, NCH_2_*CH_2_*CH_2_N), 2.76 (s, 6H, 2 × ^+^NCH_3_), 3.01 (t, ^3^J_H,H_ = 7.20 Hz, 2H, CH_2_N^+^), 4.17 (t, ^3^J_H,H_ = 7.35 Hz, 2H, CONCH_2_), δ 7.40-7.43 (m, 3H, 9-, 10-, 11-H), 7.53 (d, ^3^J_H,H_ = 7.70 Hz, 1H, 6-H), 8.21-8.24 (m, 2H, 1-, 8-H), 8.44 (d, ^3^J_H,H_ = 7.75 Hz, 1H, 5-H), 8.63 (d, ^3^J_H,H_ = 8.15 Hz, 1H, 2-H). ESI-Mass: calcd m/z for C_23_H_21_ClN_2_O_2_S: 424.10; found: 389.1 [M-Cl]^+^. Anal. Calcd for C_23_H_21_ClN_2_O_2_S: C, 65.01; H, 4.98; N, 6.59; S, 7.55. Found: C, 64.85; H, 4.75; N, 6.89; S, 7.95.

N-(3-(diethylamino)propyl)benzo[*k*, *l*]thioxanthene-3,4-dicarboximide hydrochloride **S2**: Benzothioxanthene-3,4-dicarboxylic anhydride **S9** was reacted with N,N-diethyl-1,3-propanediamine according to the general procedure (c) to give the N-(3-(diethylamino)propyl)benzo[*k*, *l*]thioxanthene-3,4-dicarboximide **S2a** as a yellow red solid, yield 94.0 %, mp 188.4–190.5 °C. Then **S2a** produces **S2** in the presence of dry hydrogen chloride according to the general procedure (d) as a yellow solid. R_f_: 0.17 (DCM/methanol 12/1, v/v), yield 92.6%, mp: >300 °C. ^1^H-NMR (500 MHz, CDCl_3_): δ 1.33 (t, ^3^J_H,H_ = 7.3 Hz, 6H, 2 × CH_3_), 2.21 (m, 2H, NCH_2_*CH_2_*CH_2_N), 3.02-3.08 (m, 6H, 2 × ^+^NCH_2_ + CH_2_N^+^), 4.20 (t, ^3^J_H,H_ = 7.05 Hz, 2H, CONCH_2_), 7.29-7.38 (m, 4H, 6- H, 9-H, 10-H, 11-H), 7.99 (d, ^3^J_H,H_ = 8.15 Hz, 1H, 5-H), 8.06 (m, 1H, 8-H), 8.23 (d, ^3^J_H,H_ = 8.10 Hz, 1H, 1-H), 8.41 (d, ^3^J_H,H_ = 8.10 Hz, 1H, 2-H). ESI-Mass: Calcd m/z for C_25_H_25_ClN_2_O_2_S: 452.13; found: 417.2 [M-Cl]^+^. Anal. Calcd for C_25_H_25_ClN_2_O_2_S: C, 66.28; H, 5.56; N, 6.18; S, 7.08. Found: C, 66.08; H, 5.70; N, 6.39; S, 6.82.

N-(3-(Diethanolamino)propyl)benzo[*k*, *l*]thioxanthene-3,4-dicarboximide hydrochloride **S3**: Benzothioxanthene-3,4-dicarboxylic anhydride **S9** was reacted with N-(3-Aminopropyl)diethanolamine according to the general procedure (c) to give the N-(3-(Diethanolamino)propyl)benzo[*k*, *l*]thioxanthene-3,4-dicarboximide **S3a** as a yellow red solid, yield 92.0 %, mp 118.5‒120.3 °C. Then **S3a** produces **S3** in the presence of dry hydrogen chloride according to the general procedure (d) as a yellow solid. R_f_: 0.13 (DCM/methanol/acetic acid 12/1/0.2, v/v), yield 90.2%, mp: >300 °C. ^1^H-NMR (500 MHz, CD_3_OD): δ 2.26 (m, 2H, NCH_2_*CH_2_*CH_2_N), 3.03 (t, ^3^J_H,H_ = 7.25 Hz, 2H, CH_2_N^+^), 3.15 (t, ^3^J_H,H_ = 5.20 Hz, 4H, 2 × ^+^NCH_2_), 3.94 (m, 4H, 2 × *CH_2_*OH), 4.17 (t, ^3^J_H,H_ = 7.5 Hz, 2H, CONCH_2_), δ 7.26-7.41 (m, 4H, 9-, 10-, 11-H), 7.51 (d, ^3^J_H,H_ = 7.95 Hz, 1H, 6-H), 8.20-8.23 (m, 2H, 5-, 8-H), 8.42 (d, ^3^J_H,H_ = 8.10 Hz, 1H, 1-H), 8.62 (d, ^3^J_H,H_ = 8.10 Hz, 1H, 2-H). ESI-Mass: Calcd m/z for C_25_H_25_ClN_2_O_4_S: 484.12; found: 449.2 [M-Cl]^+^. Anal. Calcd for C_25_H_25_ClN_2_O_4_S: C, 61.91; H, 5.20; N, 5.78; S, 6.61. Found: C, 61.50; H, 5.00; N, 6.98; S, 6.90.

N-(3-(pyrrolidin-1-yl)propyl)benzo[*k*, *l*]thioxanthene-3,4-dicarboximide hydrochloride **S4**: Benzothioxanthene-3,4-dicarboxylic anhydride **S9** was reacted with 3-(pyrrolidin-1-yl)propan-1-amine according to the general procedure (c) to give the N-(3-(pyrrolidin-1-yl)propyl)benzo[*k*, *l*]thioxanthene-3,4-dicarboximide **S4a** as a yellow red solid, yield 94.3 %, mp 155.7–157.9 °C. Then **S4a** produces **S4** in the presence of dry hydrogen chloride according to the general procedure (d) as a yellow solid. R_f_: 0.16 (DCM/methanol 12/1, v/v), yield 92.7%, mp: >300 °C. ^1^H-NMR (500 MHz, DMSO-d_6_): δ 1.59 (m, 4H, CH_2_*CH_2_CH_2_*CH_2_), 1.80 (t, ^3^J_H,H_ = 7.20 Hz, 2H, NCH_2_*CH_2_*CH_2_N), 2.42-2.44 (m, 4H, 2 × ^+^NCH_2_), 2.65 (t, ^3^J_H,H_ = 7.30 Hz, 2H, CH_2_^+^N), 4.12 (t, ^3^J_H,H_ = 7.40 Hz, 2H, CONCH_2_), 7.48-7.54 (m, 2H, 9-, 10-H), 7.59-7.61 (m, 1H, 11-H), 7.75 (d, ^3^J_H,H_ = 8.10 Hz,1H, 6-H), 8.33 (d, ^3^J_H,H_ = 8.10 Hz, 1H, 5-H), 8.45-8.51 (m, 3H, 1-, 2-, 8-H). ESI-Mass: Calcd m/z for C_25_H_23_ClN_2_O_2_S: 450.12; found: 415.3 [M-Cl]^+^, 344.3 [M-NC_4_H_9_Cl]^+^. ESI-Mass: Calcd m/z for C_25_H_23_ClN_2_O_2_S: 450.12; found: 431.1 [M-Cl]^+^. Anal. Calcd for C_25_H_23_ClN_2_O_2_S: C, 66.58; H, 5.14; N, 6.21; S, 7.11. Found: C, 66.34; H, 5.30; N, 6.20; S, 7.26.

N-(3-(piperidin-1-yl)propyl)benzo[*k*, *l*]thioxanthene-3,4-dicarboximide hydrochloride **S5**: Benzothioxanthene-3,4-dicarboxylic anhydride **S9** was reacted with 3-(piperidin-1-yl)propan-1-amine according to the general procedure (c) to give the N-(3-(piperidin-1-yl)propyl)benzo[*k*, *l*]thioxanthene-3,4-dicarboximide **S5a** as a yellow red solid, yield 94.5 %, mp 167.5‒169.8 ℃. Then **S5a** produces **S5** in the presence of dry hydrogen chloride according to the general procedure (d) as a yellow solid. R_f_: 0.18 (DCM/methanol 12/1, v/v), yield 91.5%, mp: >300 ℃. ^1^H-NMR (500 MHz, CD3OD): δ 1.53-2.21 (m, 8H, CH_2_*CH_2_CH_2_CH_2_*CH2 + NCH_2_*CH_2_*CH_2_N), 2.98 (t, ^3^J_H,H_ = 10 Hz, 2H, CH_2_N^+^), 3.23-3.25 & 3.59-3.62 (m, 4H, 2 × ^+^NCH_2_), 4.22 (t, ^3^J_H,H_ = 7.50 Hz, 2H, CONCH_2_), 7.39-7.47 (m,4H, 6-, 9-, 10-, 11-H), 8.17 (d, ^3^J_H,H_ = 7.5 Hz, 1H, 5-H), 8.21-8.24 (m, 2H, 1-, 8-H), 8.40 (d, ^3^J_H,H_ = 8.00 Hz, 1H, 2-H). ESI-Mass: Calcd m/z for C_25_H_25_ClN_2_O_2_S: 464.13; found: 429. [M-Cl]^+^. Anal. Calcd for C_25_H_25_ClN_2_O_2_S: C, 67.16; H, 5.42; N, 6.02; S, 6.90. Found: C, 67.34; H, 5.21; N, 5.87; S, 7.21.

N-(3-morpholinopropyl)benzo[*k*, *l*]thioxanthene-3,4-dicarboximide hydrochloride **S6**: Benzothioxanthene-3,4-dicarboxylic anhydride **S9** was reacted with 3according to the general procedure (c) to give the N-(3-morpholinopropyl)benzo[*k*, *l*]thioxanthene-3,4-dicarboximide **S6a** as a yellow red solid, yield 94.0 %, mp 165.7–168.1 ℃. Then **S6a** produces **S6** in the presence of dry hydrogen chloride according to the general procedure (d) as a yellow solid. R_f_: 0.15 (DCM/methanol 12/1, v/v), yield 92.8%, mp: >300 ℃. ^1^H-NMR (500 MHz, CDCl_3_): δ 1.94-1.97 (m, 2H, NCH_2_*CH_2_*CH_2_N), 2.46 (m, 4H, 2 × ^+^NCH_2_), 2.53 (t, ^3^J_H,H_ = 7.05 Hz, 2H, CH_2_N^+^), 3.64 (t, ^3^J_H,H_ = 4.50 Hz, 4H, 2 × CH_2_O), 4.27 (t, ^3^J_H,H_ = 7.35 Hz, 2H, CONCH_2_), δ 7.40-7.44 (m, 3H, 9-, 10-, 11-H), 7.52 (d, 1H, ^3^J_H,H_ = 8.00 Hz, 6-H), 8.20-8.23 (m, 2H, 5-, 8-H), 8.43 (d, ^3^J_H,H_ = 8.00 Hz, 1H, 1-H), 8.62 (d, ^3^J_H,H_ = 8.10 Hz, 1H, 2-H). ESI-Mass: Calcd m/z for C_25_H_23_ClN_2_O_3_S: 466.11; found: 431.1 [M-Cl]^+^. Anal. Calcd for C_25_H_23_ClN_2_O_3_S: C, 64.30; H, 4.96; N, 6.00; S, 6.87. Found: C, 64.60; H, 5.20; N, 6.32; S, 6.43.


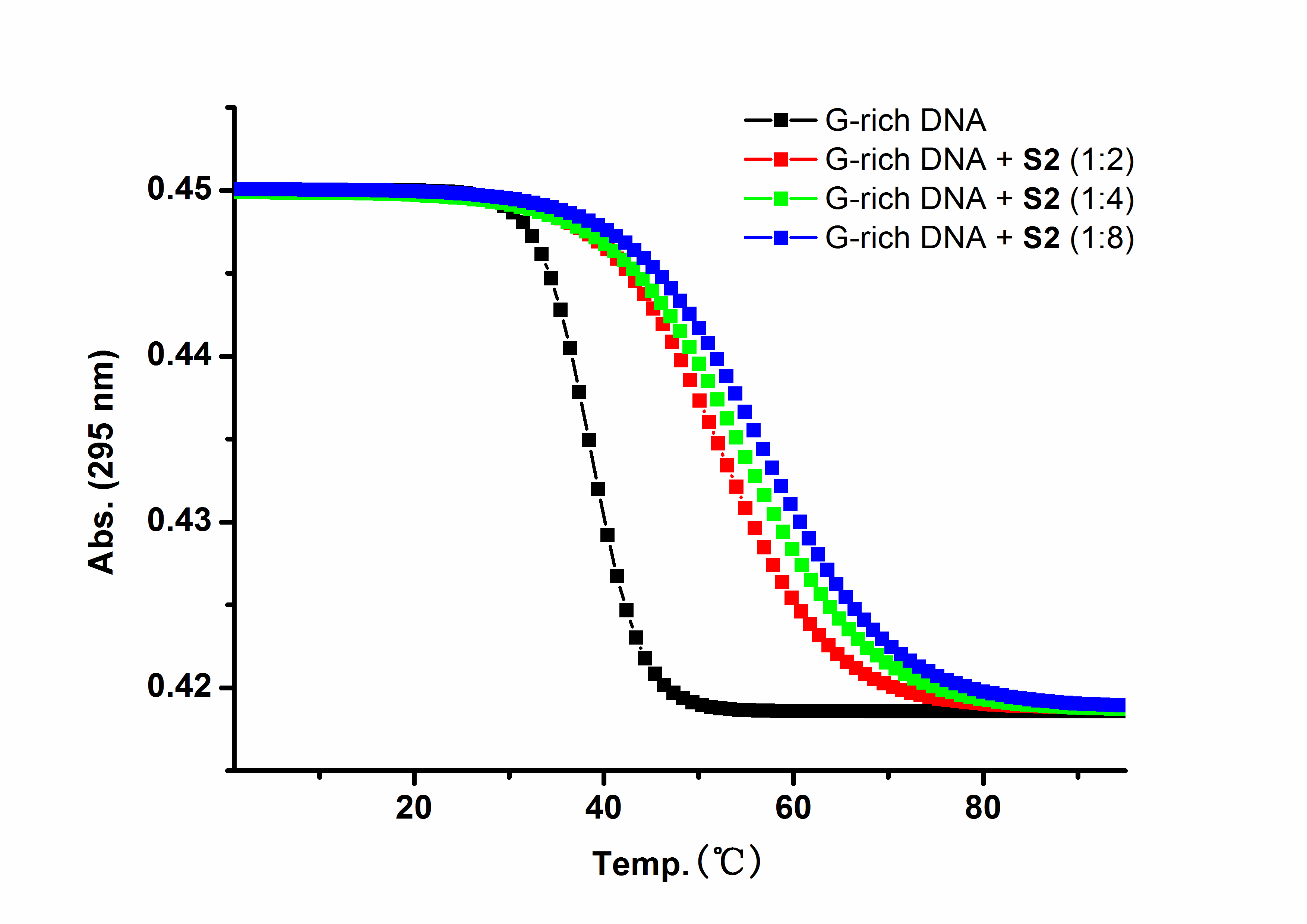

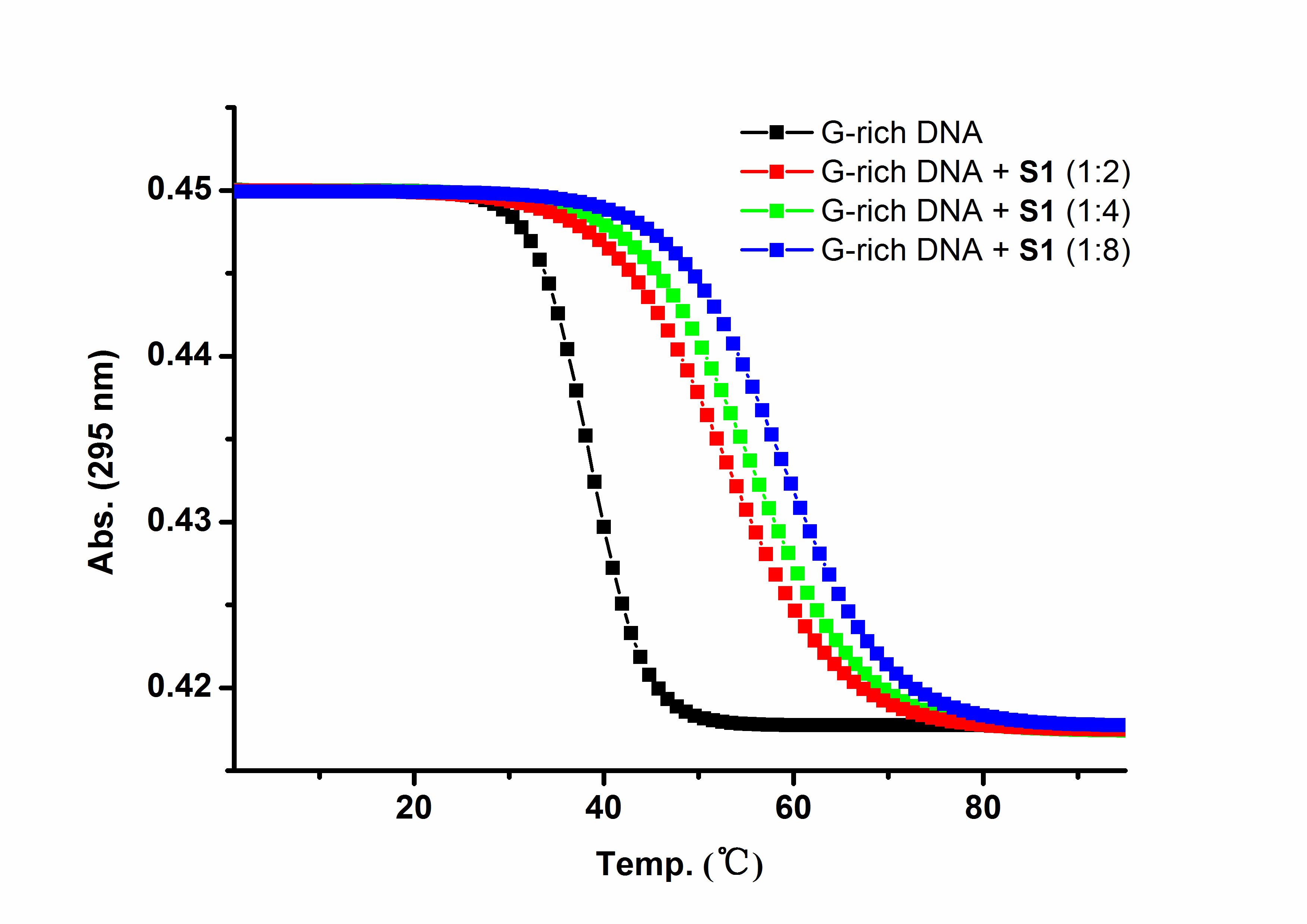

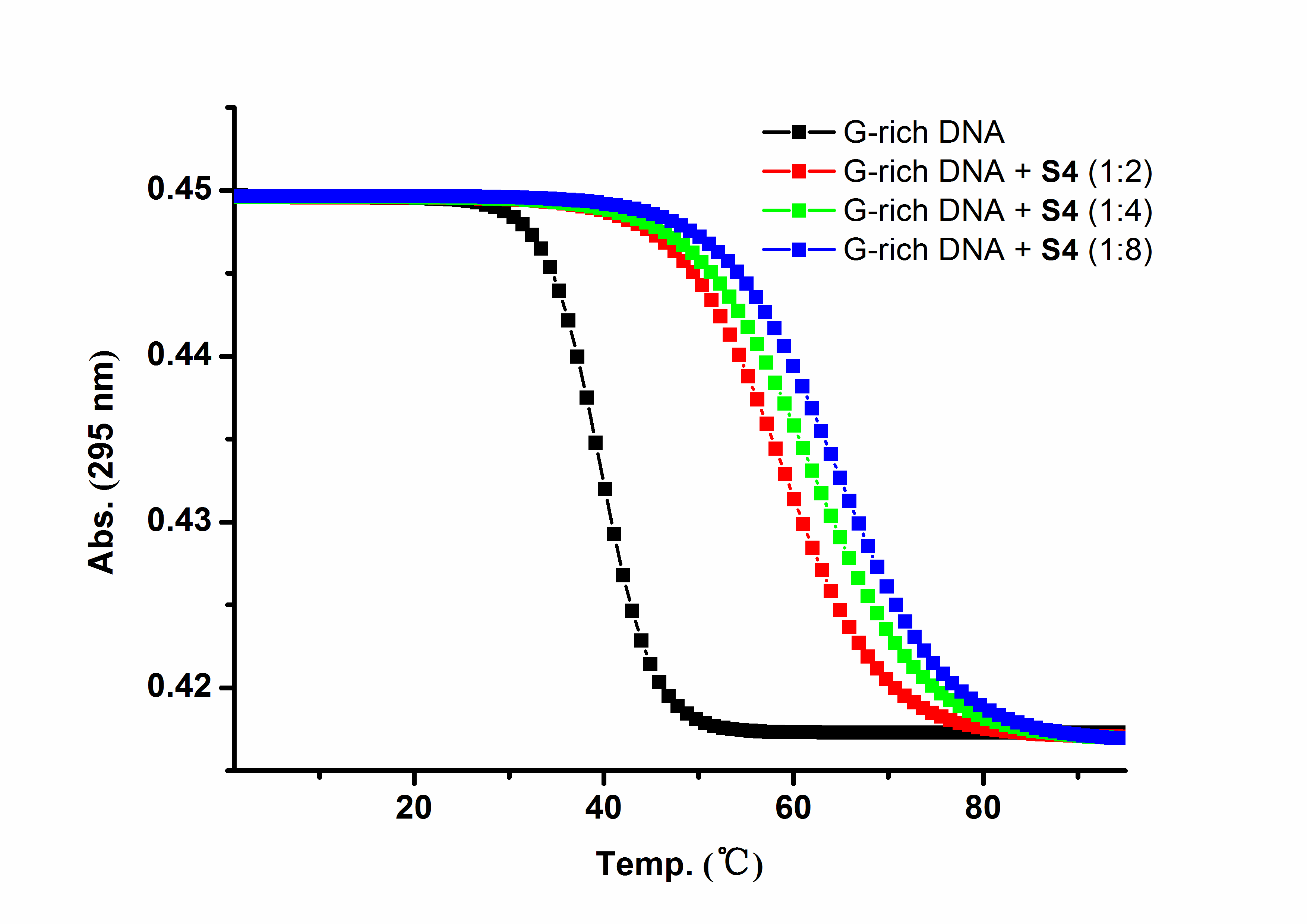

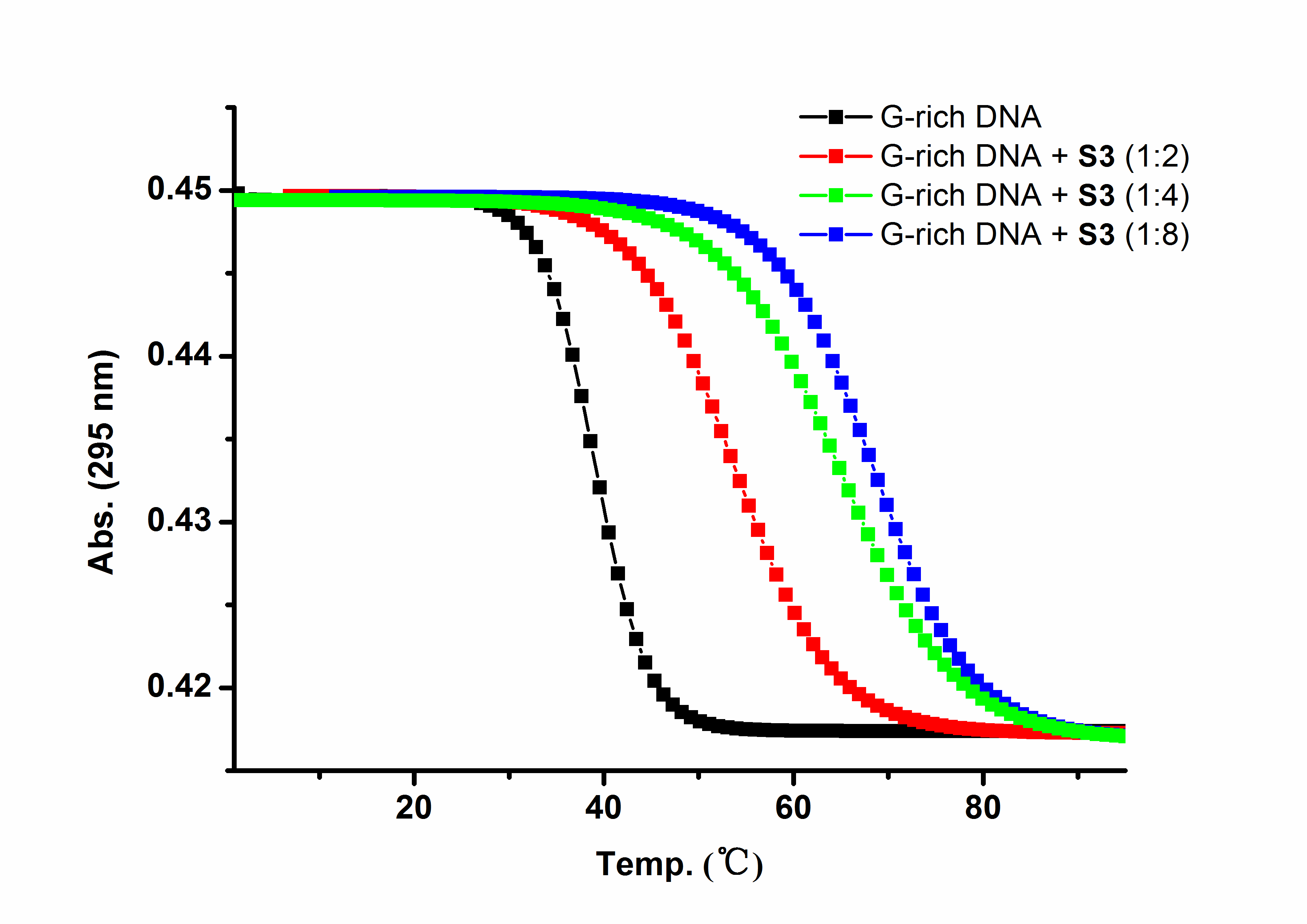

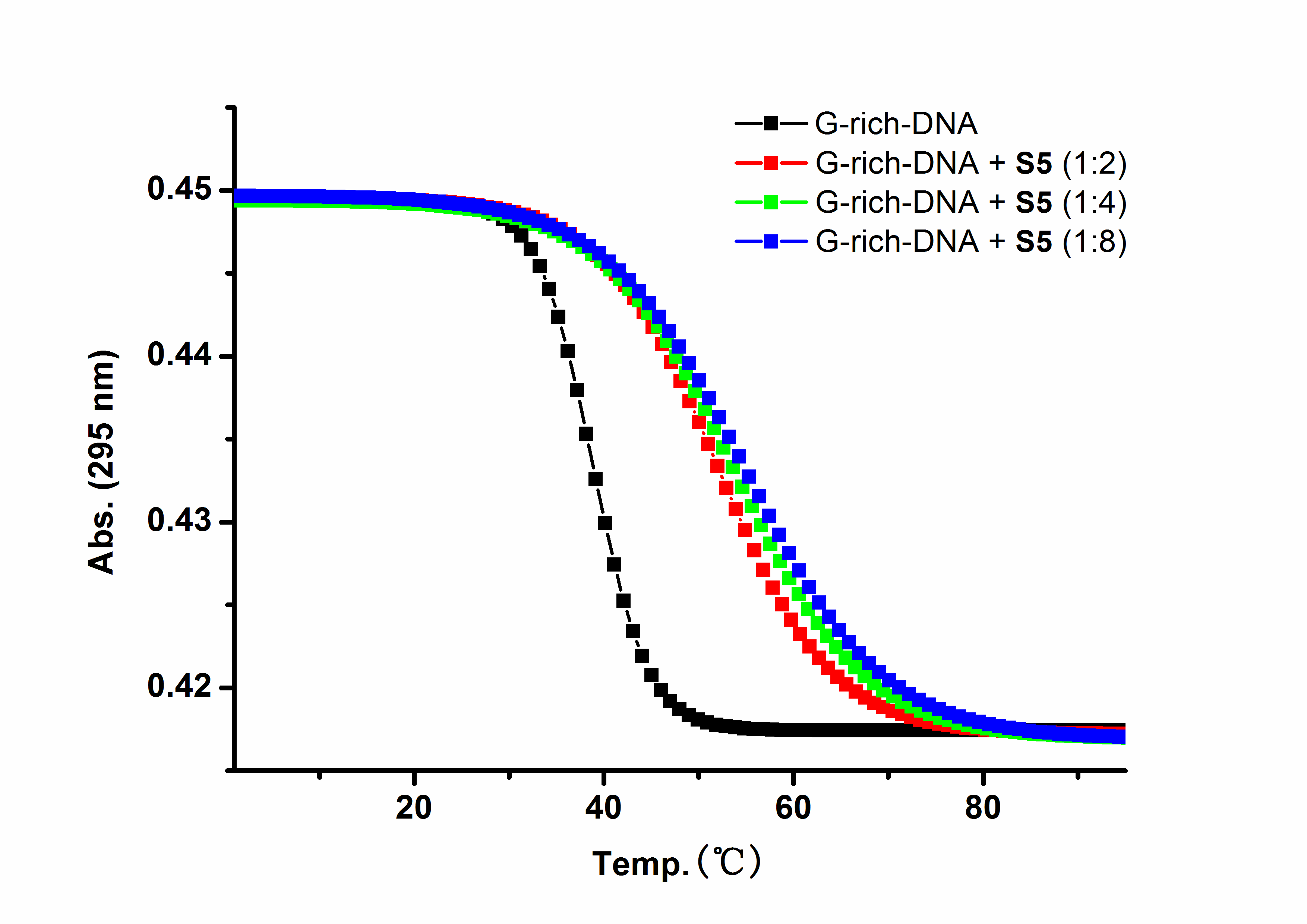

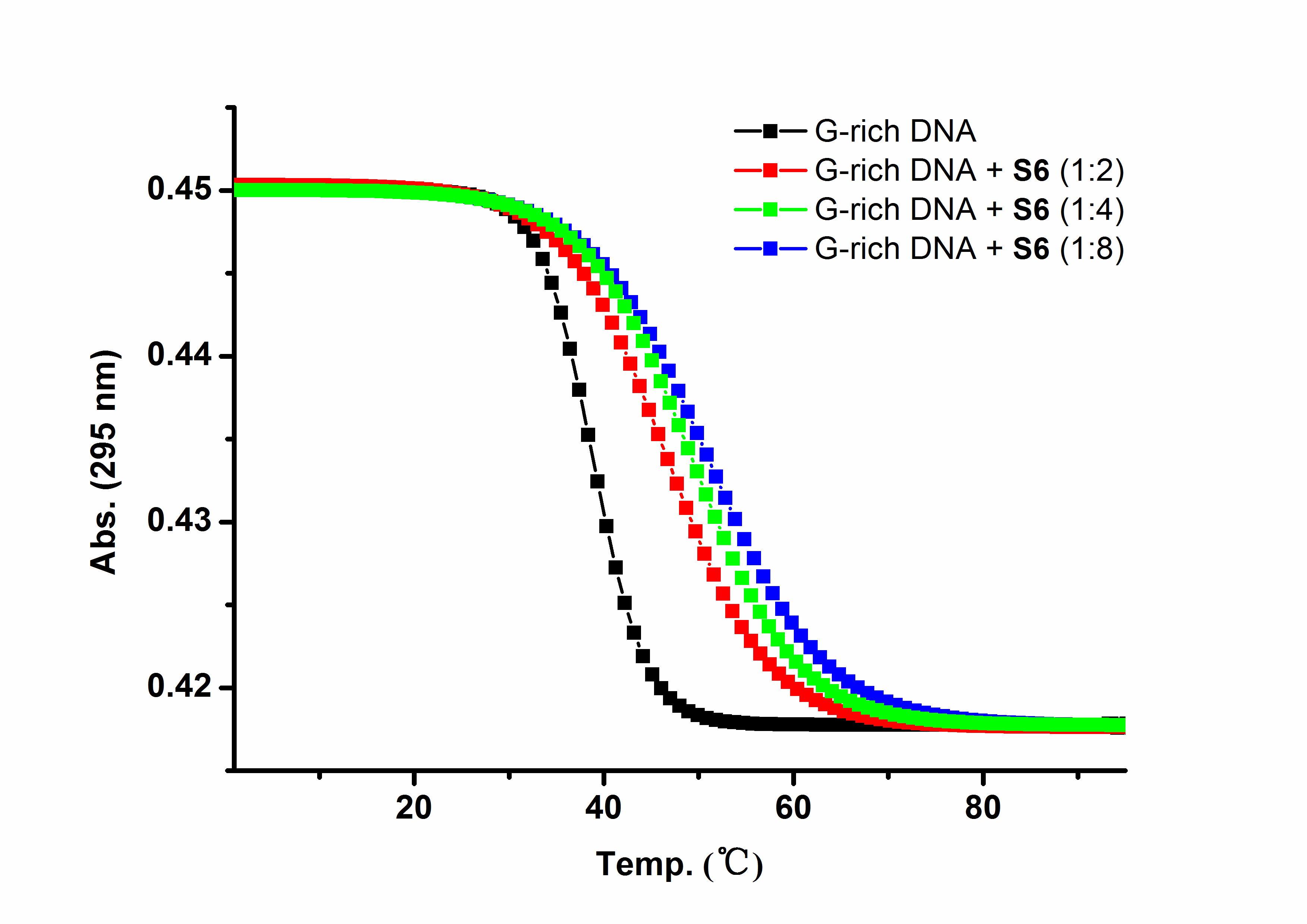


**A**

**B**

**C**

**D**

**E**

**F**

**Figure S2. UV-melting curves for the interaction between benzothioxanthene derivatives S1 (A), S2 (B), S3 (C), S4 (D), S5 (E), S6 (F) and the 26nt telomeric G-rich DNA sequence of human chromosome, respectively, in 10 mM K^+^, incubated for 24h.** All experiments were performed in a buffer (pH 7.4) containing 10 mM Tris–HCl, 10 mM KCl, 0.1 mM EDTA, and samples of 2 μM DNA dissolved in the buffer in the presence and absence of compounds **S1**-**S6** were incubated for 24 h after annealing at 95 °C. The absorbance for resulting mixtures was monitored at the temperatures form 0 °C through 95 °C. The curve shows the melting profiles. The ordinate shows the relative absorbance of the samples. The abscissa shows the temperature in °C. For the G-rich DNA, the curves correspond to compound concentrations of 0, 4, 8, and 16 μM, increasing from left to right. Using UV-melting analysis system, shape analysis of the melting curves yielded transition temperatures (*T_m_*), which are the midpoint temperatures of the helix–coil transitions. The cell chamber was continuously flushed with nitrogen gas to prevent water condensation at low temperatures. The melting profiles for the quardruplex formed by the 26nt G-rich DNA were acquired at 295 nm.

**A**

**
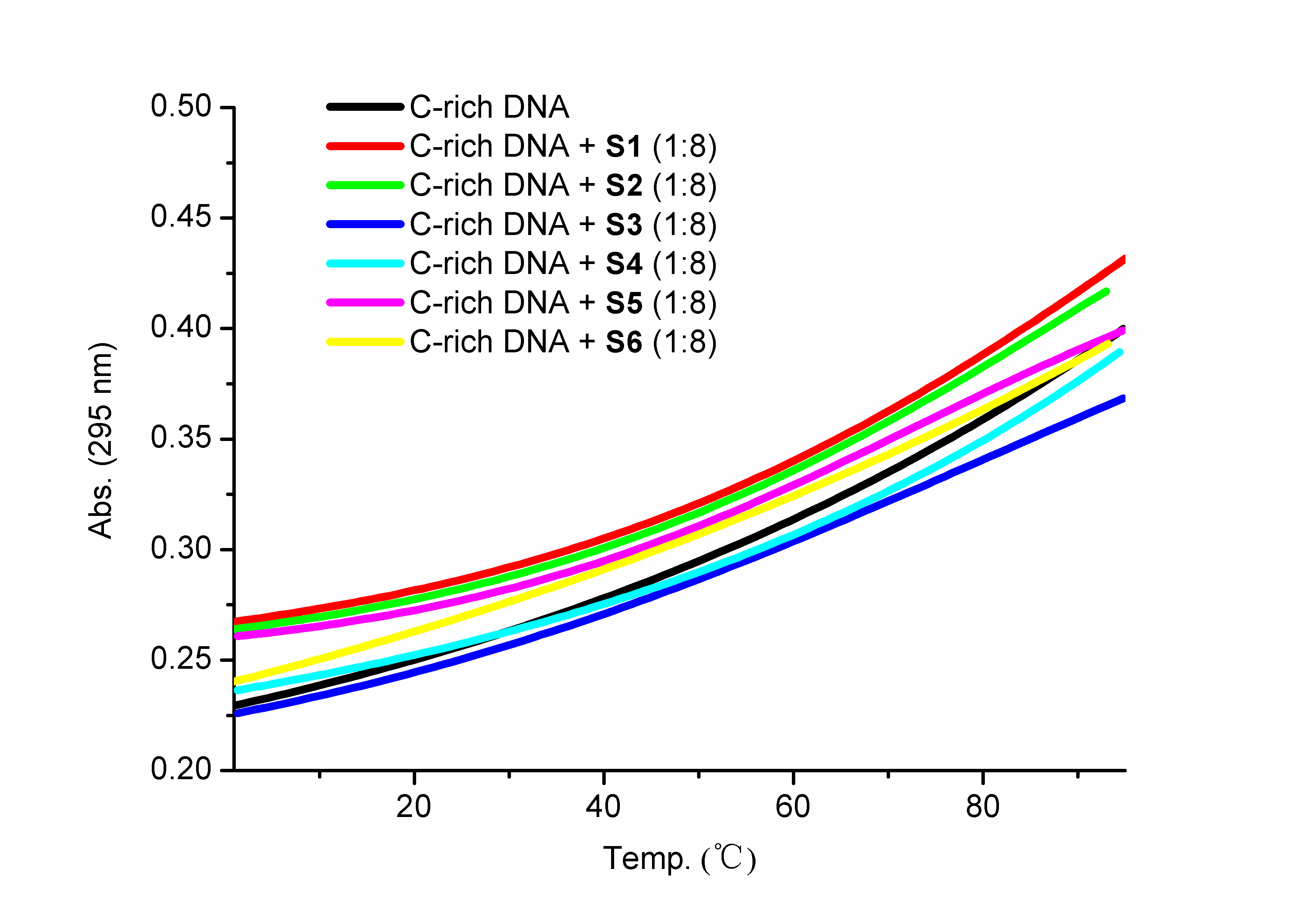
**

**B**

**
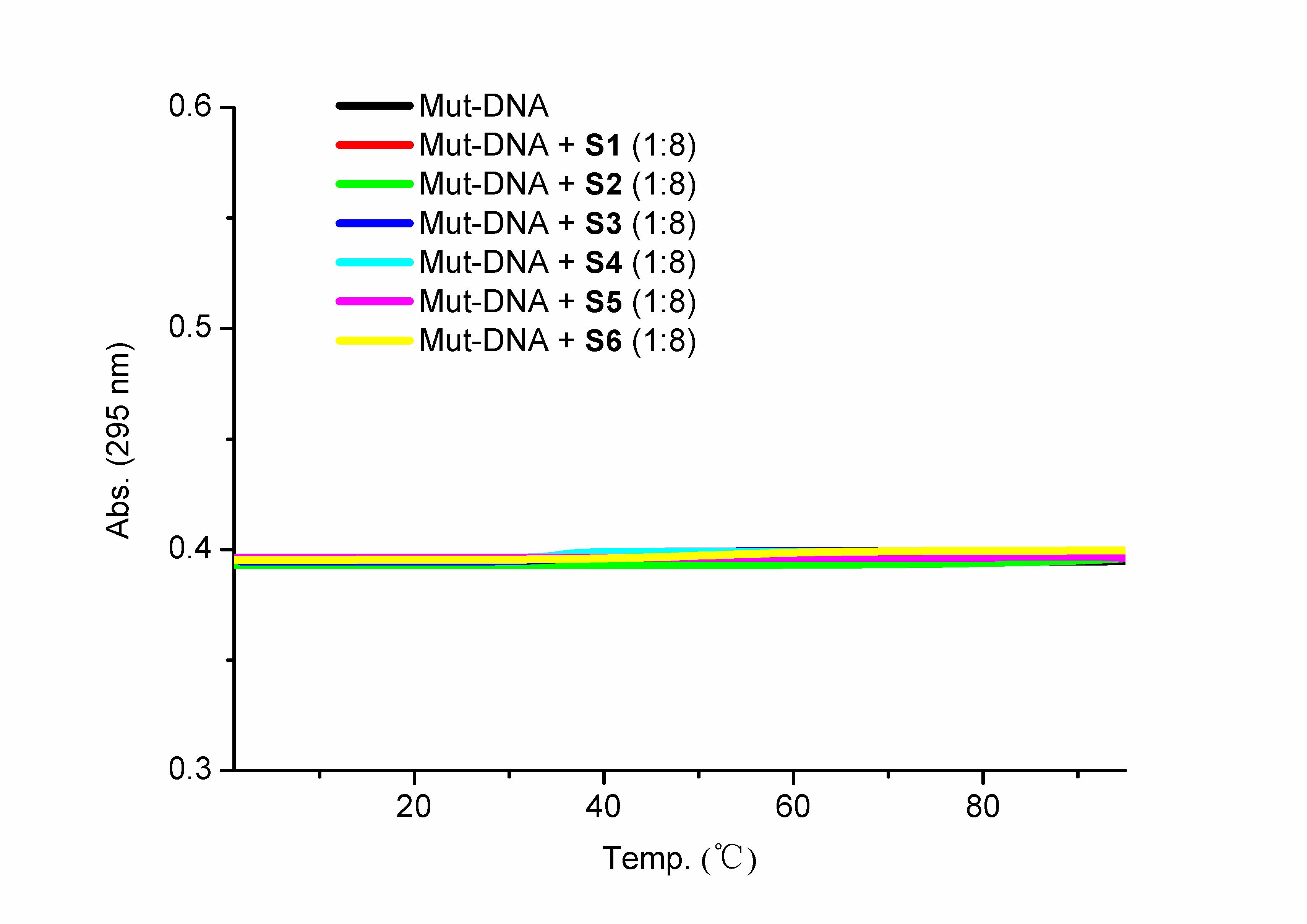
**

C

**
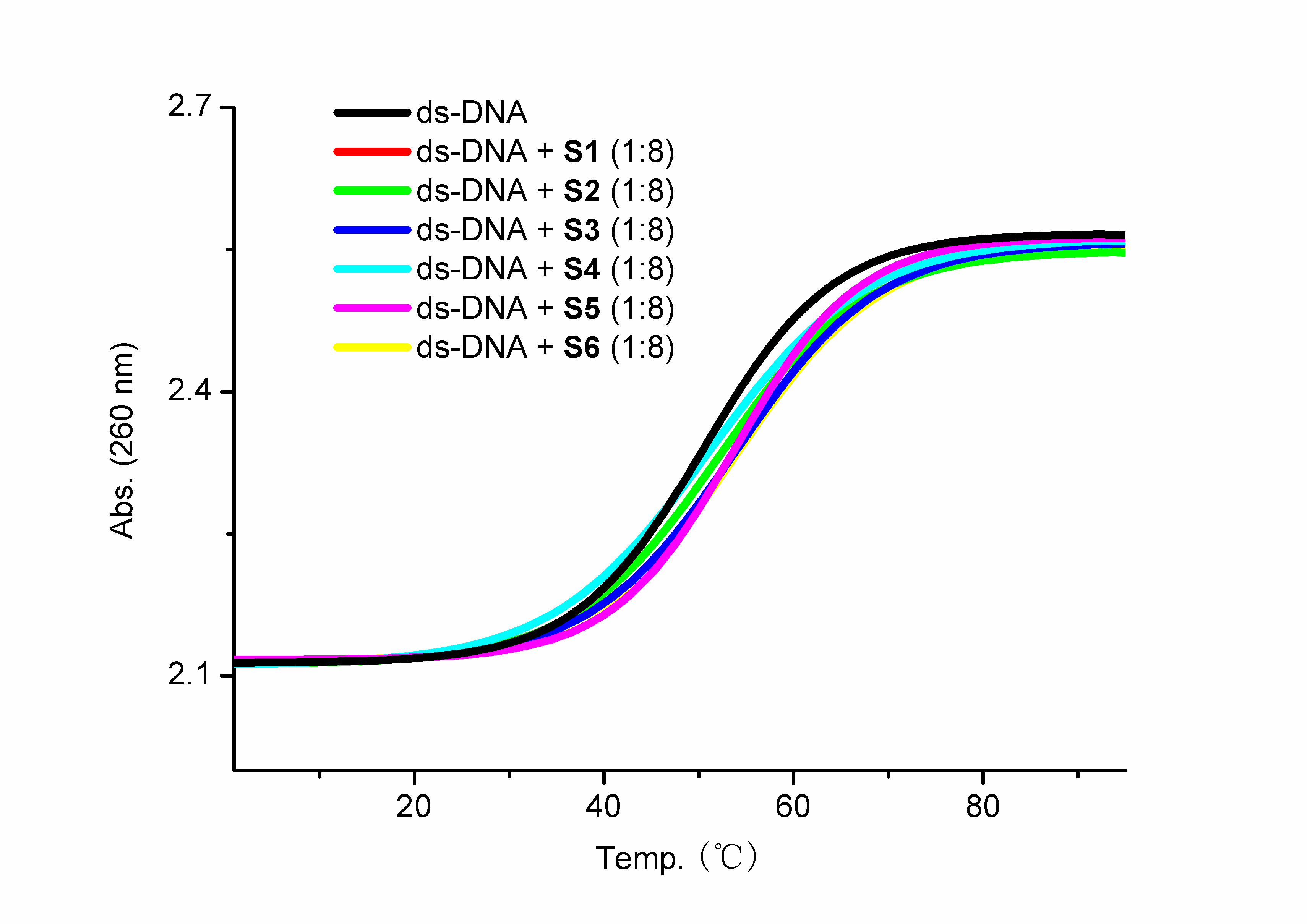
**

**Figure S3. UV-melting curves for the interaction between benzothioxanthene derivatives and 26nt telomeric (for C-rich DNA (A), Mut-DNA (B), ds-DNA (C) sequences of human chromosome in 10 mM K^+^, incubated for 24h.** All experiments were performed in a buffer (pH 7.4) containing 10 mM Tris–HCl, 10 mM KCl, 0.1 mM EDTA, and samples of 2 μM DNA dissolved in the buffer were incubated for 24 h after annealing at 95 °C**.** The curve shows the melting profiles. The ordinate shows the relative absorbance of the samples. The abscissa shows the temperature in °C. For C-rich-DNA (**A**), Mut-DNA (**B**) and ds-DNA (**C**), the compound concentration was 0, 16 μM. The melting profiles for the quardruplex formed by single-stranded-DNA were acquired at 295 nm, while the melting profiles for ds-DNA were acquired at 260 nM.

**A.** C-rich DNA + **S1**


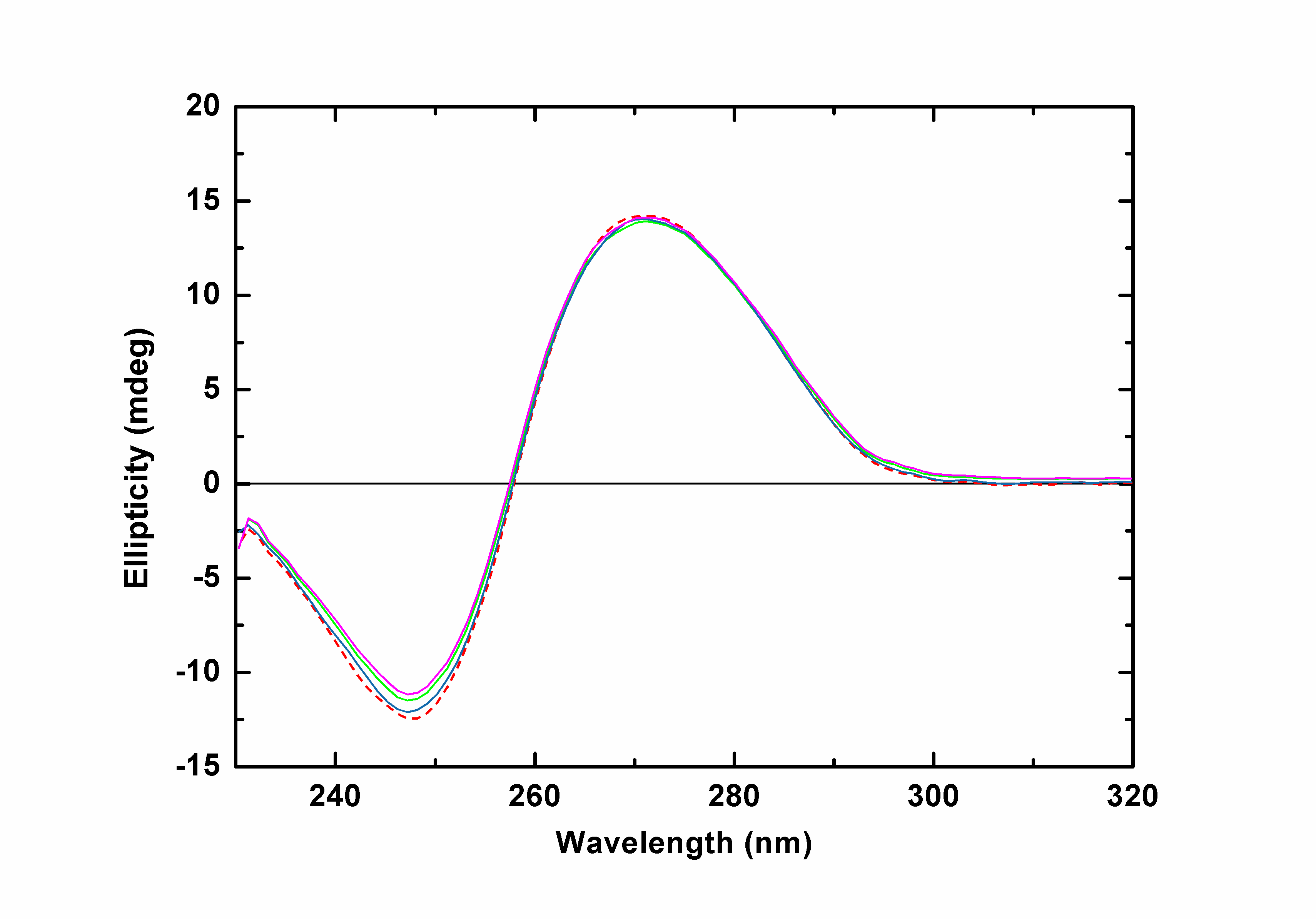


**B.** C-rich DNA + **S3**


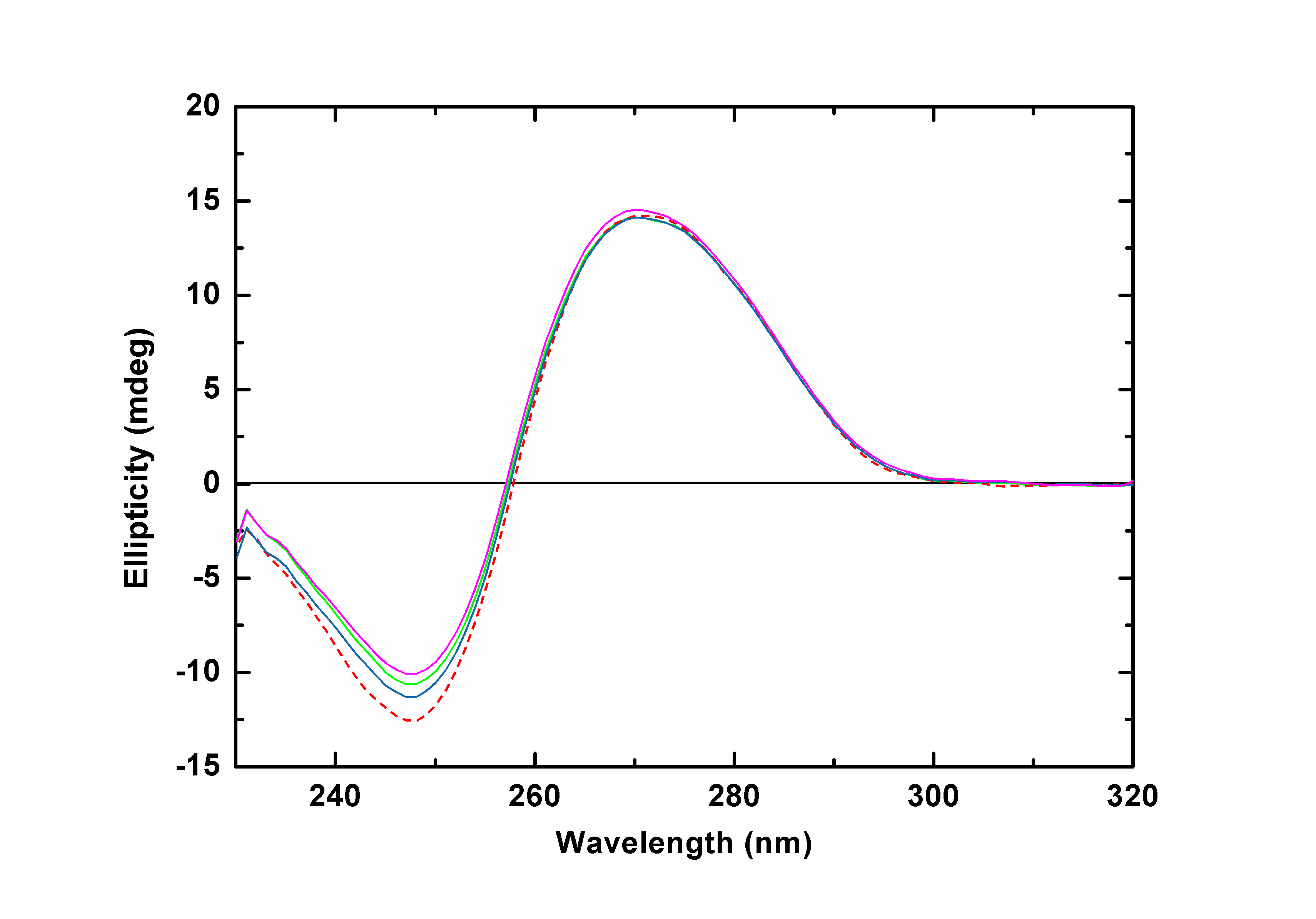


**C.** C-rich DNA + **S4**


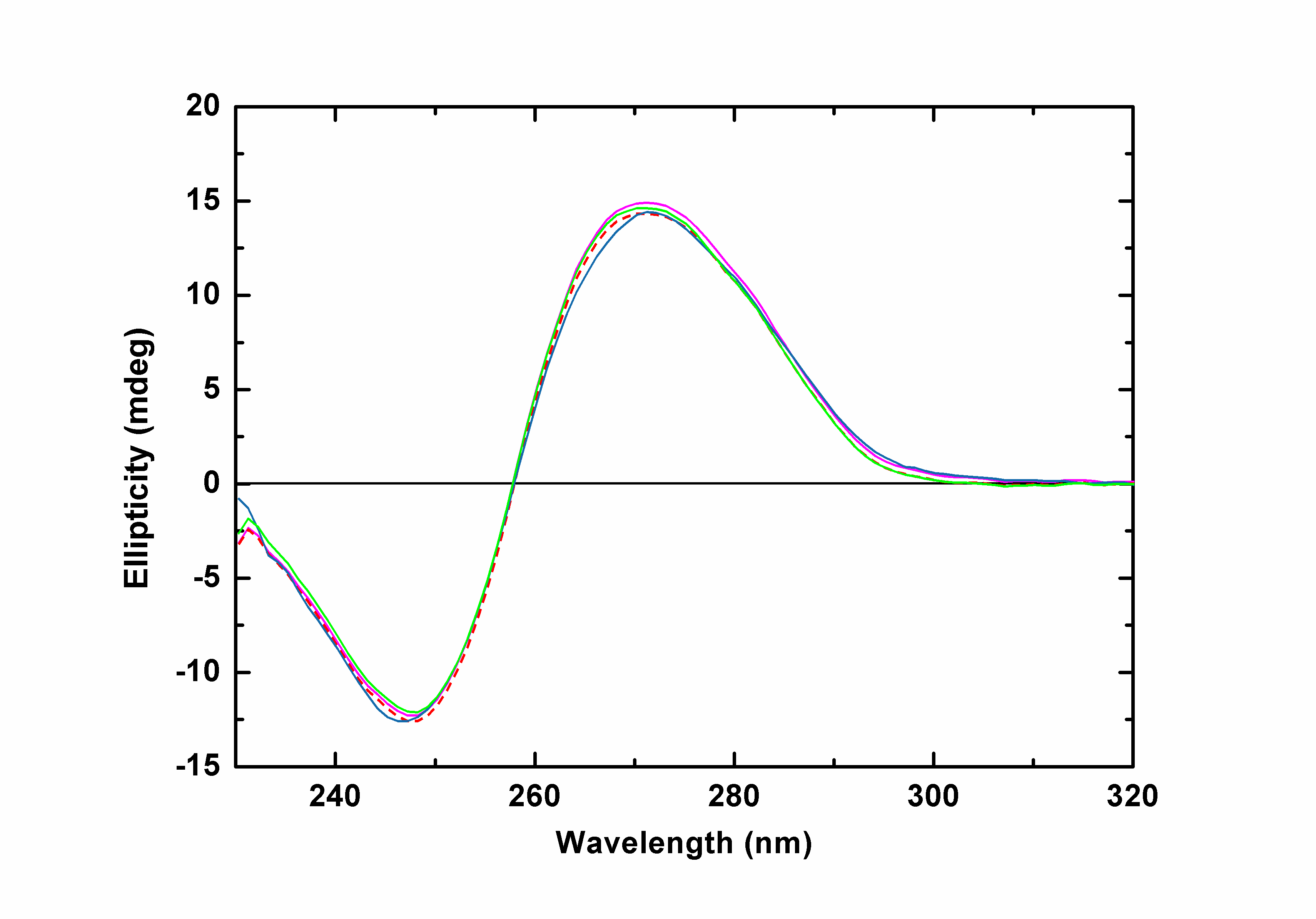


**D.** Mut-DNA + **S1**

**
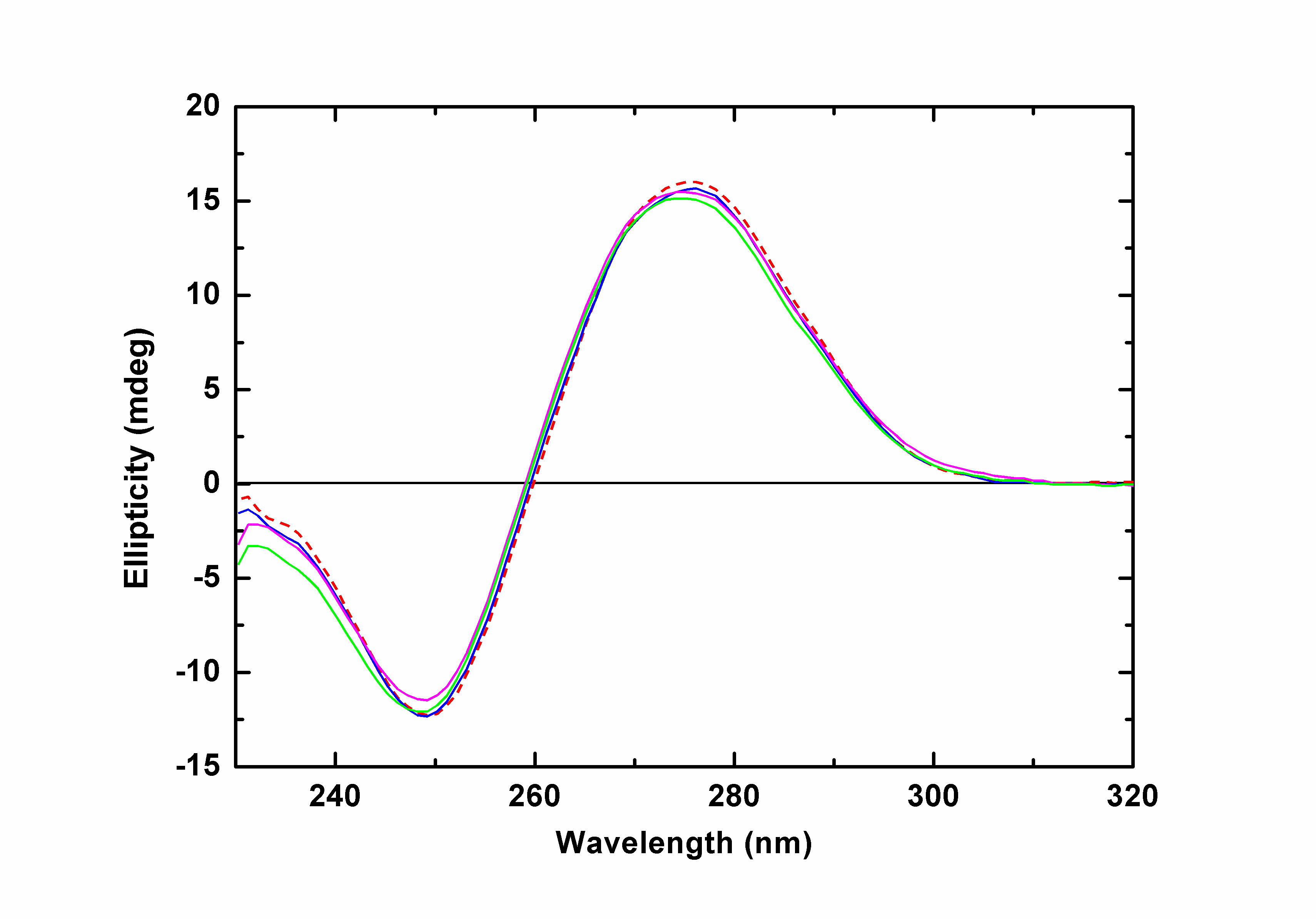
**

**E.** Mut-DNA + **S3**

**
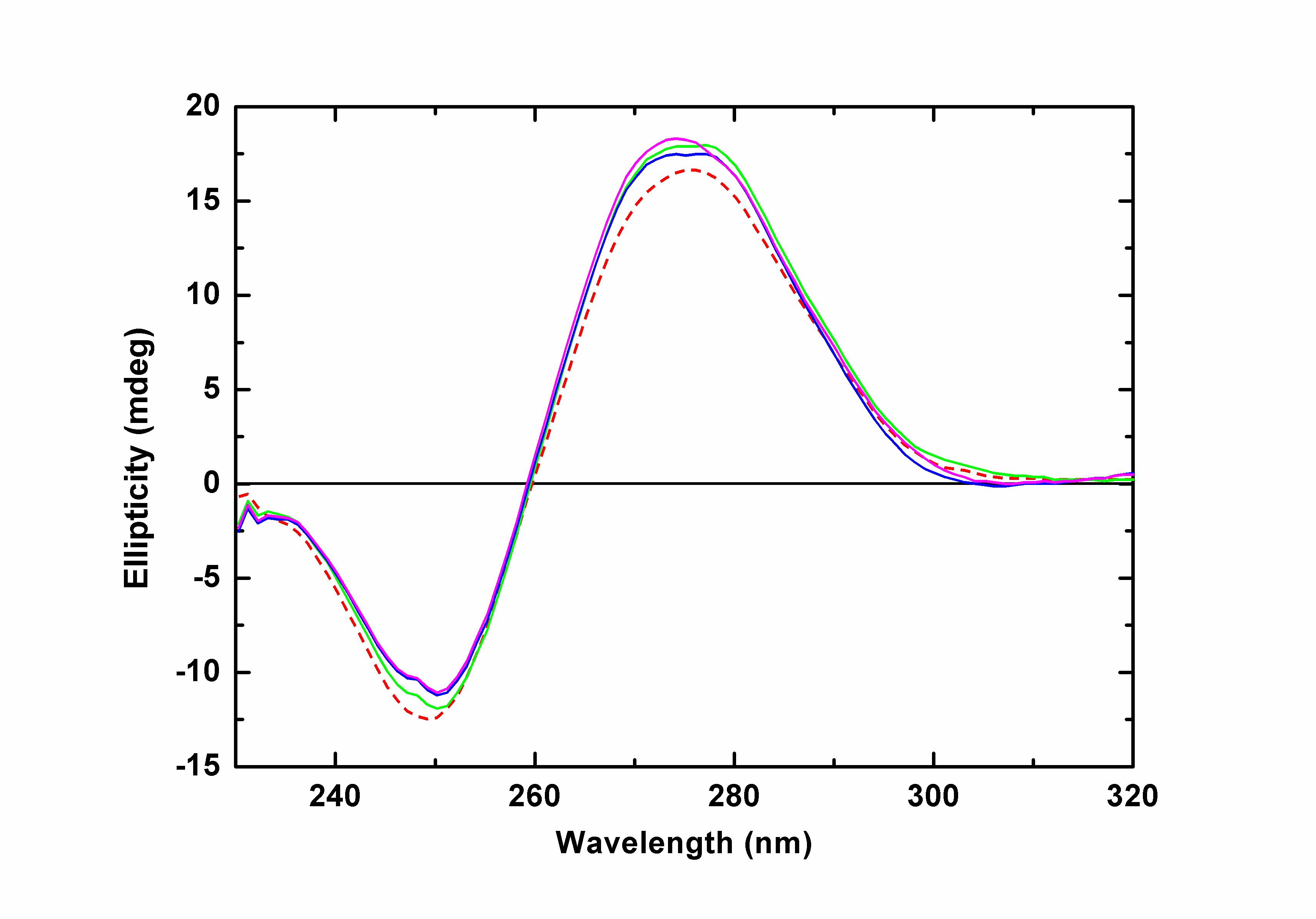
**

**F.** Mut-DNA + **S4**

**
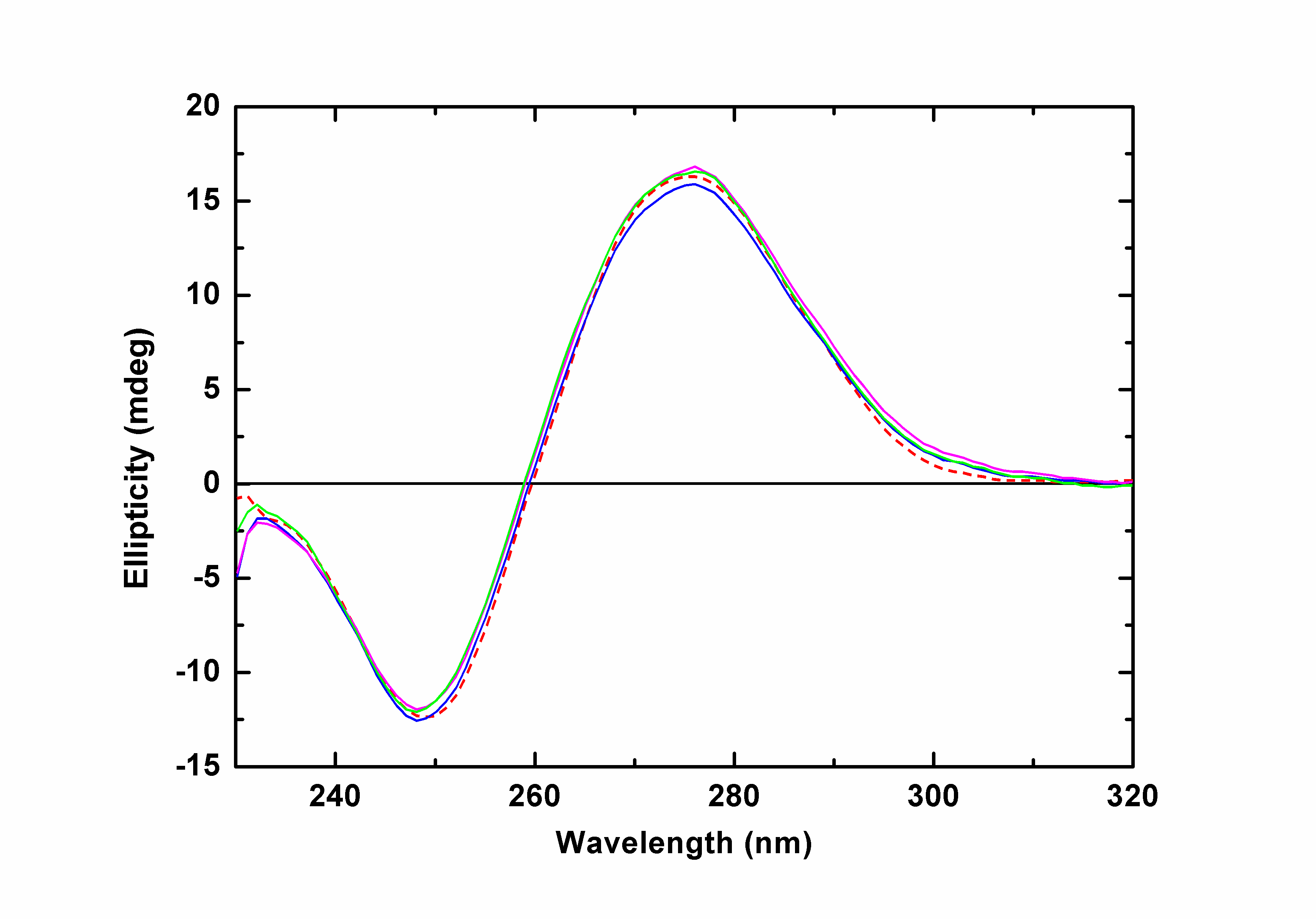
**

**G.** ds-DNA + **S1**

**H.** ds-DNA + **S4**

**Figure S4. CD spectra for the interaction between benzothioxanthene derivatives S1, S3, S4 and 26nt telomeric/mutated DNA sequence of human chromosome in 10 mM K^+^ by titration of S1, S3 and S4 into DNA, respectively.** All experiments were performed in a buffer (pH 7.4) containing 10 mM Tris–HCl, 10 mM KCl, 0.1 mM EDTA, and samples of 4 μM C-rich/Mut- DNAs (in single strand) and 2 μM double-stranded DNA (in double strand) dissolved in the buffer were incubated for 24 h after annealing at 95 °C**.** The CD spectra shown from 320-230 nm were obtained in the presence/absence of compounds at the incremental concentrations of 0 (red dash), 4 μM (blue solid line), 20 μM (green solid line), 40 μM (violet solid line ) for C-rich/Mut- DNAs (**A**-**F**) and 0 (red dash), 2 μM (blue solid line), 8 μM (green solid line), 32 μM (violet solid line ) for ds-DNA (**G**, **H**), respectively.


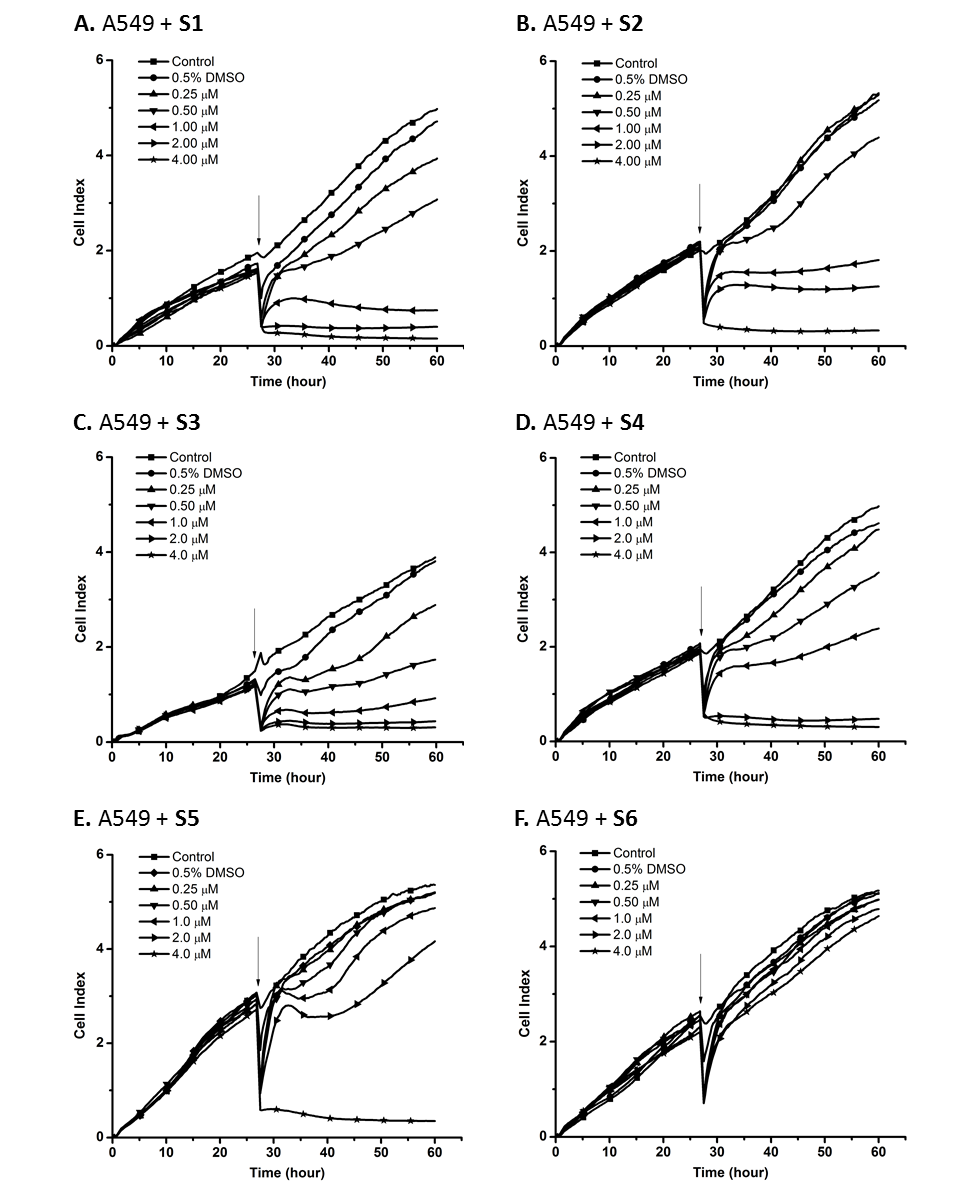


**Figure S5. The cell index curve obtained by Real-time cell analysis (RTCA) reflecting the apoptosis of A549 cells in the presence/absence of S1 (A), S2 (B), S3 (C), S4 (D), S5 (E), S6 (F) at increased concentrations of 0.25, 0.50, 1.0, 2.0, 4.0 μM.** 0.5% DMSO and cell culture medium alone were as a reference and a negative control, respectively. Arrows indicate time point of compound addition. Different compound concentrations and references are shown by different line plus symbols. Data are normalized to the time of compound addition at 24 hr of cell culture. Each trace is typical of 3 replicates.


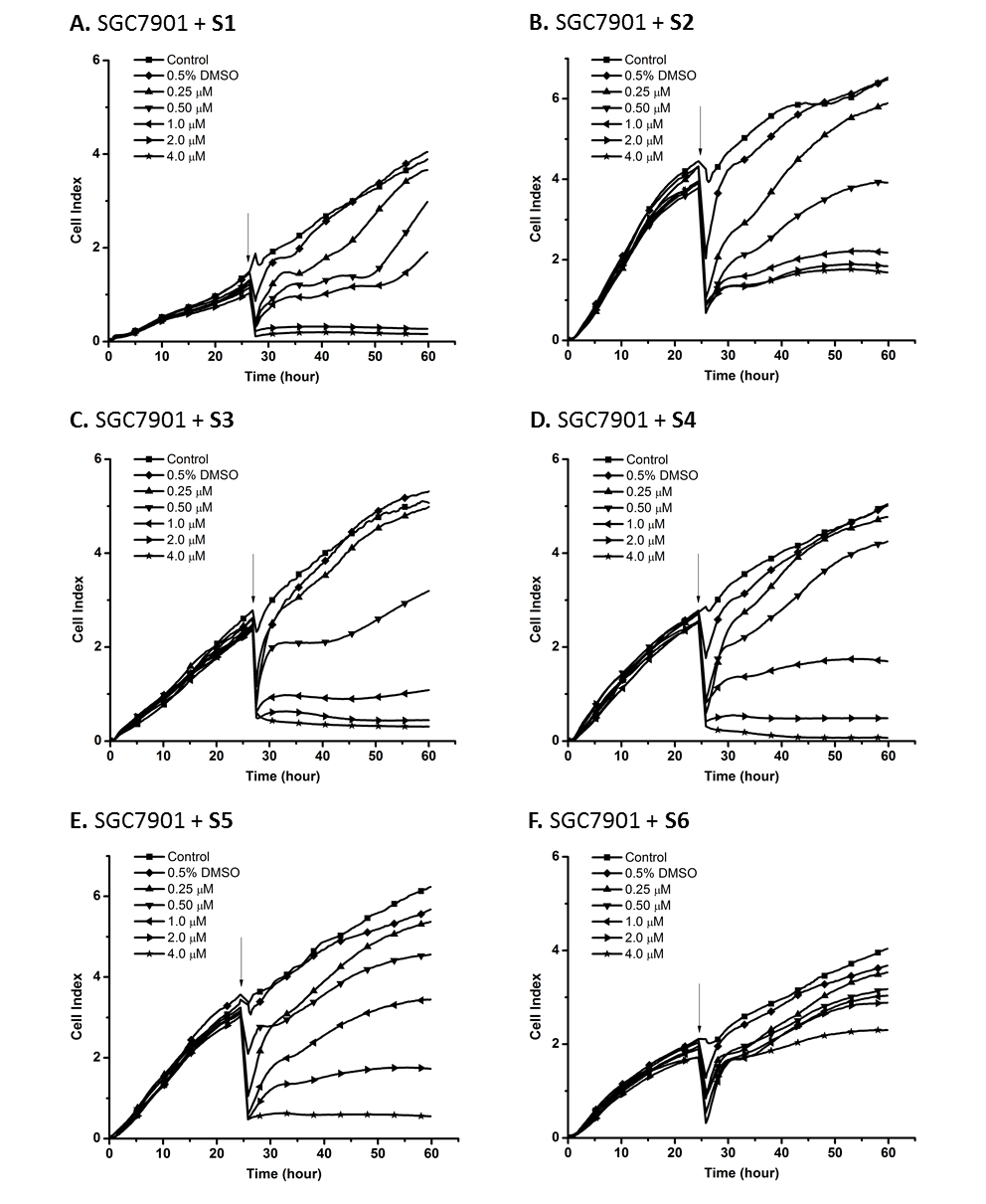


**Figure S6. The cell index curve obtained by Real-time cell analysis (RTCA) reflecting the apoptosis of SGC7901 cells in the presence/absence of S1 (A), S2 (B), S3 (C), S4 (D), S5 (E), S6 (F) at increased concentrations of 0.25, 0.50, 1.0, 2.0, 4.0 μM.** 0.5% DMSO and cell culture medium alone were as a reference and a negative control, respectively. Arrows indicate time point of compound addition. Different compound concentrations and references are shown by different line plus symbols. Data are normalized to the time of compound addition at 24 hr of cell culture. Each trace is typical of 3 replicates.


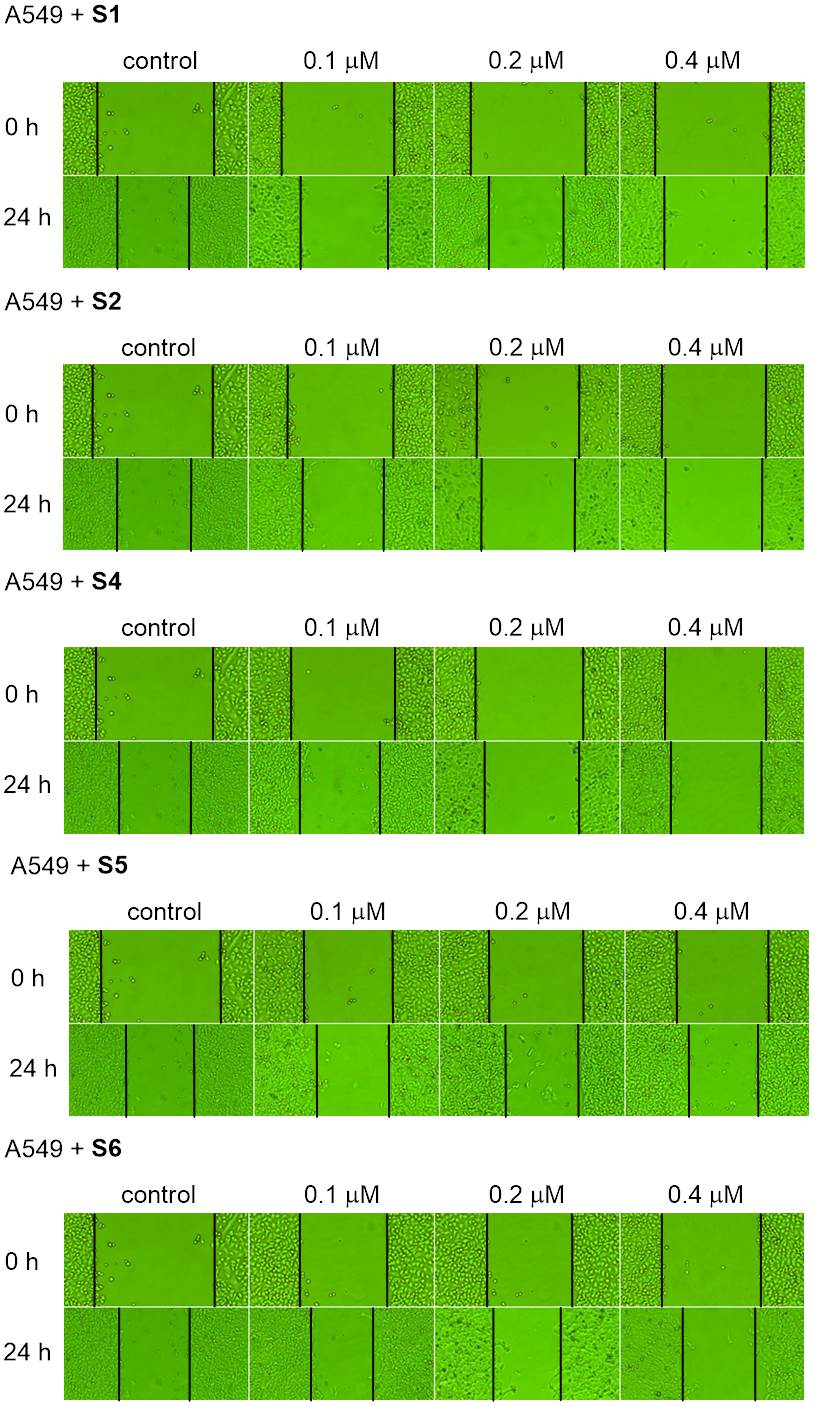


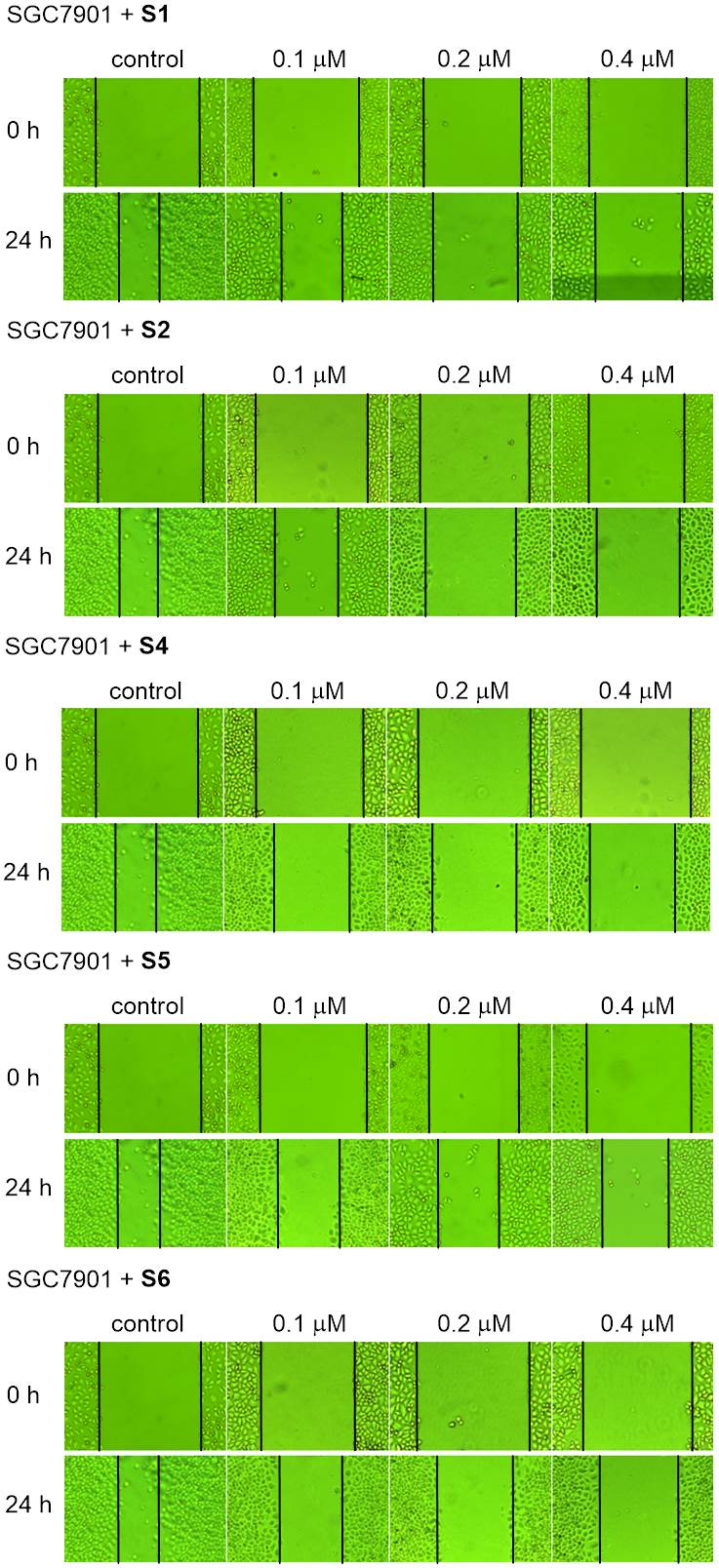


**Figure S7. Effect of benzothioxanthene derivatives on the mobility of tumor cells.** A549 and SGC7901 cells were grown in culture dish and streaked with a pipet chip. After the addition of **S1**, **S2**, **S4**, **S5**, or **S6** to the culture at the indicated concentrations, mobility of the cells was recorded under a microscope. The width of the streaked line was measured at four to six reference points along the originally streaked line. Representative results of three independent experiments in triplicates were shown (P＜0.05).

**Reference**

1. Grayshan, P. H., Kadhim, A. M. & Perters A. T. Heterocyclic derivalives of naphthalene-1,8-dicar boxylie anhydride. Part III. Benzo[*k,l*] thioxanthene-3,4-dicarboximides. *J. Heterocyclic Chem.* [**11**,](http://onlinelibrary.wiley.com/doi/10.1002/jhet.v11:1/issuetoc)33–38 (1974).
